# Preferences and beliefs about financial risk taking mediate the association between anterior insula activation and self-reported real-life stock trading

**DOI:** 10.1038/s41598-018-29670-6

**Published:** 2018-07-25

**Authors:** Alexander N. Häusler, Camelia M. Kuhnen, Sarah Rudorf, Bernd Weber

**Affiliations:** 1 Center for Economics and Neuroscience, University of Bonn, Nachtigallenweg 86, 53127 Bonn, Germany; 2 Department of Epileptology, University Hospital Bonn, Sigmund-Freud-Strasse 25, 53127 Bonn, Germany; 3 Department of NeuroCognition/Imaging, Life&Brain Research Center, Sigmund-Freud-Strasse 25, 53127 Bonn, Germany; 4 Kenan-Flagler Business School, University of North Carolina, 300 Kenan Center Drive, Chapel Hill, NC 27599 USA; 5 Department of Social Psychology and Social Neuroscience, Institute of Psychology, University of Bern, Fabrikstrasse 8, 3012 Bern, Switzerland

## Abstract

People differ greatly in their financial risk taking behaviour. This heterogeneity has been associated with differences in brain activity, but only in laboratory settings using constrained behaviours. However, it is important to understand how these measures transfer to real life conditions, because the willingness to invest in riskier assets has a direct and considerable effect on long-term wealth accumulation. In a large fMRI study of 157 working age men (39.0 ± 6.4 SD years), we first show that activity in the anterior insula during the assessment of risky vs. safe choices in an investing task is associated with self-reported real-life active stock trading. We then show that this association remains intact when we control for financial constraints, education, the understanding of financial matters, and cognitive abilities. Finally, we use comprehensive measures of preferences and beliefs about risk taking to show that these two channels mediate the association between brain activation in the anterior insula and real-life active stock trading.

## Introduction

Functional magnetic resonance imaging (fMRI) has been used extensively to study financial risk taking in the laboratory, but less work has connected brain activation to financial risk taking choices in real life^1–6^. Here, we use data from a non-student sample to study a self-reported real-life indicator of financial risk taking that has a significant impact on wealth accumulation, namely, whether people trade stocks. In addition, we measure brain activation during an investing task and study the differences in the estimated mean activation of these brain regions between active stock traders and non-active stock traders (our binary dependent variable “active stock trading” was assessed using question 21 in the Supplementary Document “Do you trade stocks yourself?”, see Supplementary Information). Previous studies have consistently linked the neural implementation of decisions under risk and, more broadly, value based choices to activity in brain regions such as the ventral striatum (VS), the anterior insula (AI), and the ventromedial prefrontal cortex (vmPFC)^1–12^. Even though these studies have greatly improved our knowledge of the neural mechanisms underlying risky choices^13^, it is unknown how the observed heterogeneity in individual brain activity and behaviour in laboratory tasks transfers to financial risk taking in real life^12,14^.

More specifically, studies of financial decision making under risk have linked risk seeking behaviour to VS activation, while risk averse choices have been shown to relate to increased activation in the AI^1,2,4–6^. Recent efforts have additionally shown that the interaction between these two regions plays a role in making financial choices under risk^11,12^. In our investing task, participants are repeatedly asked to choose between a risky option (stock) and a safe option (bond) while being in either a gain or loss domain. We expect that individuals who trade stocks in real life show higher VS and lower AI activation during a risky versus a safe option. Furthermore, we believe that the chance to find these differences in VS and AI activation between active and non-active stock traders is higher in the gain domain, due to this part of the investing paradigm being more similar to stock trading in real life (in the gain domain of the investing task participants try to win money, while in the loss domain they try to avoid losses). With regard to the established role of the vmPFC in subjective value and reward processing^3,9,10,15^, we hypothesize that individuals who trade stocks show higher vmPFC activation upon choosing the stock versus the bond and receive higher reward-related vmPFC activation upon getting the feedback to have made the correct choice after having chosen the risky versus the safe option. In a sample of 157 working age men, we find that, out of the three candidate regions (VS, AI, and vmPFC), only activation in the AI when choosing a risky over a safe option in the gain domain of an investing task is significantly lower in participants who actively trade stocks in real life, compared with those who do not.

Next, we investigate possible economic mechanisms by which the AI activation may influence real-life financial risk taking. Due to the fact that stock market participation has been associated with financial constraints^16,17^, we assess whether higher AI activation in people who do not trade stocks may simply reflect these individuals’ financial constraints and their interest to avoid further financial risk. We do not find this to be the case, because AI activation continues to be associated with real-life stock trading, even after we control for participants’ household income and financial liabilities. We then inquire whether AI activation could be a proxy for education, as well as a general understanding of financial matters and cognitive abilities, because all these factors have been found to relate to stock market participation^16–22^. We do not find that these individual characteristics influence the association between the AI activation and real-life active stock trading. Next, we assess whether people’s preferences and beliefs about risk taking^14,23,24^ are captured by the AI activation differences between active and non-active stock traders. We use a combination of self-assessment questions and behavioural data from decisions under risk to create two comprehensive characteristics that evaluate people’s beliefs regarding the outcomes of risky choices (risk optimism index (ROI)) and the willingness to bear risk (risk tolerance index (RTI)). Here, we find that the association between real-life active stock trading and AI activation is mediated by both of these channels.

Our paper contributes to the neuroeconomics literature by showing that brain activation measured in the laboratory is associated with financial risk taking in real life, thus lending external validity to previous laboratory studies. We show this by using brain regions of interest from previous neuroeconomic landmark studies^1,6,9^ to identify differences in brain activation between groups of active stock traders and non-active stock traders during a stock investing task. Our paper then additionally contributes to the field of neuroeconomics by showing that differences in brain activation do not capture differences in terms of financial or cognitive constraints between these two groups of individuals, but that the association between real-life financial risk taking and AI activation is significantly mediated by comprehensive, independent measures of people’s beliefs about risk taking, as well as their risk tolerance. Hence, our results provide novel evidence that this specific brain area shown previously to relate to financial taking risk in the laboratory plays a central role for risk taking in real-life by aggregating individuals’ optimism and risk preferences.

## Results

### The fMRI paradigm is reliable from both a neuroscientific and behavioural point of view

**Figure 1:**
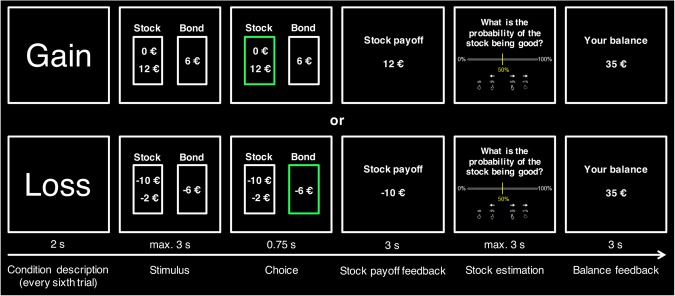
The investing paradigm and regions of interest. There were eight blocks in each domain (gain and loss), consisting of eight trials each (thus 96 total trials, one trial of each domain is shown here). In the beginning of a block, participants were shown in which domain they were. They chose between a risky (stock) or non-risky (bond) option and the choice implementation (button press) was highlighted with a green frame. Next, participants saw the stock payoff feedback, regardless of the previous choice. The participants then estimated the probability of the stock being good and finally, a balance feedback was shown.

**Figure 2:**
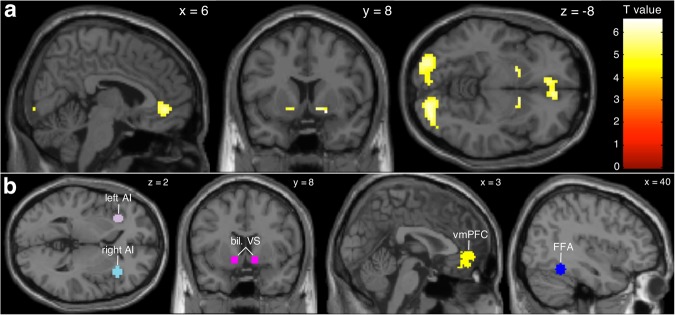
Group-level brain activation and regions of interest. (**a**) Whole-brain reward prediction error (RPE) activation (whole-brain corrected p(FWE) <0.05, k > 10, n = 165). (**b**) Regions of interest used to extract weighted beta estimates (see Figs 2–4, as well as Supplementary Figs S1, S2, and Supplementary Table S4). The right (turquoise) and left (pink) anterior insula (AI) masks, as well as bilateral ventral striatum (VS, magenta) masks were obtained from previous neuroeconomic studies on risk processing^1,6^. The ventromedial prefrontal cortex (vmPFC) mask (yellow) and the fusiform face area (FFA) mask (blue, used as a control region) were obtained from meta-analyses of valuation^9^ and emotional face processing^31^, respectively.

To measure heterogeneity in brain activity across 157 working age participants (39.0 ± 6.7 SD years), we adapted a recently established investing paradigm^25^ to a functional magnetic resonance imaging (fMRI) setting (Fig. 1). In this paradigm, we asked participants repeatedly to choose between a risky option (stock) and a safe option (bond) while being in either the gain or the loss domain (please see Experimental Design in the Methods section for more details). After each choice and regardless of the chosen option, the payoff of the stock was shown. To assess the reliability of this fMRI stock investing paradigm in relation to previous literature^2,9,10,15,26–30^, we first created a general linear model (GLM) that specifically included a parametric reward prediction error (RPE) analysis (see Supplementary Table S1). Here, we found RPE-related brain activation in the VS and the vmPFC (Fig. 2a). We then grouped participants into active stock traders and non-active stock traders according to their self-reports (see question 21 in the Supplementary Document “Do you trade stocks yourself?”, see Supplementary Information) and found that active stock traders chose the stock in the task significantly more often than non-active stock traders (first trial of each block, two-sample t-test, p = 0.029, mean choice of stock versus bond, active stock traders: 63.0 ± 27.1% SD, non-active stock traders: 53.0 ± 25.7% SD, n = 157).

### Brain activation is associated with real-life active stock trading

**Figure 3:**
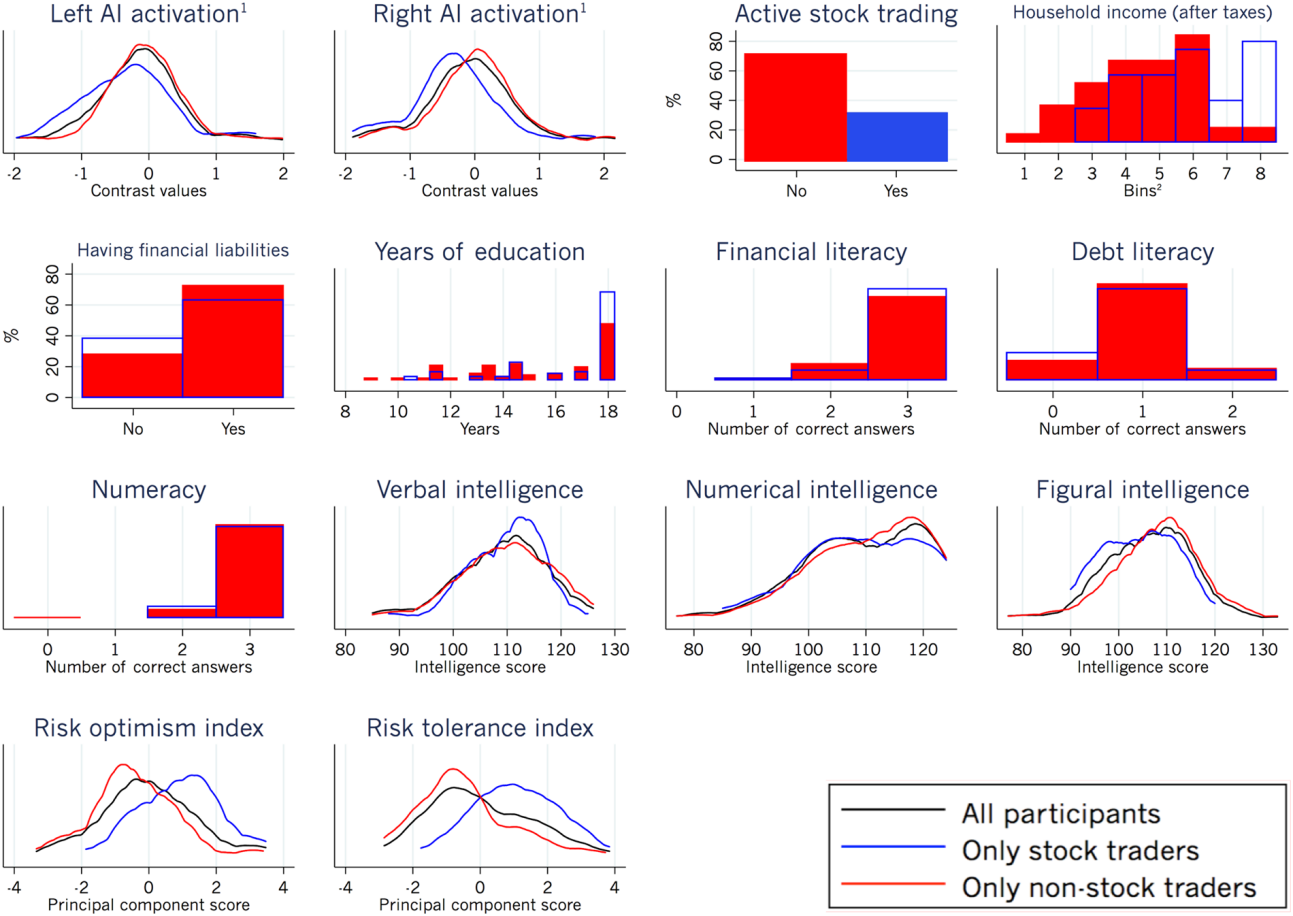
Data distribution of all variables used in the analysis (all n = 157, Tables 1–3). Kernel density graphs are shown for continuous variables, histograms for ordinal and interval variables, and bar graphs for binary variables. ^1^During stock versus bond choice (gain domain). ^2^Bins: 1 (<500€), 2 (500€–1000€), 3 (1000€–2000€), 4 (2000€–3000€), 5 (3000€–4000€), 6 (4000€–5000€), 7 (5000€–6000€), 8 (>6000€).

We then investigated whether the risk-related brain activation would help to explain real-life stock trading. Extending prior literature^1–3,6,9,12,14^, we discovered that activation in an area that was previously linked to risk averse behaviour in the lab^1,6^ – namely, the anterior insula (AI) – is a strong and significantly associated explanatory variable of people’s reluctance to trade stocks in real life. To study this association, we created a GLM (see Supplementary Table S2) that was designed to assess individual brain activation during the decision process and payoff feedback in the gain and the loss domain, separately (an overview of the whole-brain analysis is given in Supplementary Table S3). Using this GLM, we extracted the individual mean brain activation (beta estimates) in the brain regions of interest (AI, VS, and vmPFC) with masks from previous landmark studies on risk processing and valuation (Fig. 2b^1,6,9^). Additionally, we included another area with no obvious relation to valuation or risk taking (fusiform face area (FFA; Fig. 2b)) to control for specificity of effects^31^. The weighted beta estimates from these regions of interest were henceforth extracted from brain contrasts in both the choice and the payoff feedback phase (e.g., stock vs. bond choice in the gain domain, see Supplementary Table S4; distribution plots are shown in Fig. 3 and Supplementary Figs S1 and S2). Two-sample t-tests were then performed to test for brain activation differences between active and non-active stock traders. We found that activity in the VS, vmPFC, and FFA in the choice and the payoff feedback phases were not significantly different between the two groups. However, activity in the bilateral AI when participants opted for the stock vs. the bond in the gain domain was significantly associated with real-life financial risk taking (for the right AI: p = 0.0264; for the left AI: p = 0.0072; see Supplementary Table S4). Specifically, when participants opted for the stock vs. the bond in the gain domain, the AI showed lower activity in real-life active stock traders compared with non-active stock traders, consistent with the notion of a lower risk signal^1,5–7^.

### Economic variables do not explain the link between AI activation and real-life stock trading

Next, we assessed whether previous aspects that have been related to stock market participation, namely, financial constraints^16,17^, education^16,17,22^, the understanding of financial matters^20,21^, and cognitive abilities^18,19^, would explain the association between the AI activation and real-life stock trading (Tables 1 to 3; distribution plots are shown in Fig. 3). We did not find evidence for this. We first tested whether economic financial adversity would impact the association between AI activation and real-life stock trading (Tables 2 and 3), because previous studies have suggested a link between adversity^32,33^ and neural activation. When we included the value of participants’ household income and an indicator for whether they have financial liabilities (e.g. outstanding credit card debt or a mortgage) as additional explanatory variables of active stock trading, we found that this did not influence the association between AI and real-life stock trading (Tables 2 and 3). Next, we assessed whether participants’ education, understanding of financial matters, as well as their cognitive abilities could explain the link between AI activation and active stock trading. We therefore added measures of participants’ education level, as well as their financial literacy, debt literacy, numeracy, and intelligence to our model (Tables 1 to 3). To assess intelligence, we used a measure of fluid intelligence^34^ that included questions on analogies (verbal intelligence), numerical series (numerical intelligence), and matrices (figural intelligence). When we included all these measures as possible explanatory variables, we did not find that they explained the association between AI activation and real-life trading.

**Table 1:** Descriptive statistics of the variables included in the regression analysis.

	Mean	Median	SD	N	Q^#^
Active stock trading	0.3	0	0.5	157	21
Household income (after taxes)	5.0	5	1.7	157	14
Having financial liabilities	0.7	1	0.5	157	23
Years of education	15.9	17	2.5	157	5, 6
Financial literacy	2.8	3	0.4	157	39, 40, 41
Debt literacy	0.9	1	0.5	157	42, 43
Numeracy	2.9	3	0.3	157	45, 46, 47
Verbal intelligence	109.8	110	7.8	157	n.a.
Numerical intelligence	110.3	111	10.0	157	n.a.
Figural intelligence	106.8	109	8.7	157	n.a.
Risk Optimism Index (ROI)	−0.009	−0.1	1.4	157	n.a.
Risk Tolerance Index (RTI)	−0.003	−0.3	1.5	157	n.a.

Q^#^ = number in financial, risk preference, and personality questionnaire, provided as the Supplementary Document, see Supplementary Information. n.a. = not applicable.

**Table 2:** Linear probability models with possible explanatory variables of real-life active stock trading.

	Active stock trading				
Left AI (stock > bond choice, gain domain)	−0.13 (−2.24)**	−0.13 (−2.39)**	−0.10 (−1.93)*	−0.03 (−0.62)	−0.0001 (−0.00)
Household income (after taxes)		0.10 (4.72)***	0.09 (4.54)***	0.07 (3.48)**	0.06 (3.57)***
Having financial liabilities		−0.11 (−1.54)	−0.12 (−1.60)	−0.09 (−1.24)	−0.11 (−1.60)
Years of education			0.03 (1.78)*	0.02 (1.79)*	0.02 (1.66)*
Financial literacy			0.04 (0.41)	−0.08 (−0.92)	−0.05 (−0.60)
Debt literacy			−0.01 (−0.16)	−0.08 (−1.15)	−0.09 (−1.49)
Numeracy			−0.10 (−0.81)	−0.06 (−0.57)	−0.06 (−0.57)
Verbal intelligence			0.003 (0.64)	0.002 (0.39)	0.003 (0.68)
Numerical intelligence			0.0003 (0.07)	0.002 (0.45)	0.001 (0.33)
Figural intelligence			−0.01 (−1.99)**	−0.01 (−2.63)**	−0.01 (−2.22)**
Risk Optimism Index (ROI)				0.14 (5.70)***	0.10 (3.78)***
Risk Tolerance Index (RTI)					0.09 (3.95)***
N	157	157	157	157	157
Adjusted R^2^	0.03	0.15	0.16	0.31	0.37

Left AI activation is included as an independent variable of interest. Coefficients are shown with t-statistics in parentheses.

Significance levels: *p < 0.1, **p < 0.05, ***p < 0.001.

**Table 3:** Linear probability models with possible explanatory variables of real-life active stock trading.

	Active stock trading				
Right AI (stock > bond choice, gain domain)	−0.14 (−2.72)**	−0.15 (−3.14)**	−0.14 (−2.85)**	−0.10 (−2.32)**	−0.07 (−1.58)
Household income (after taxes)		0.10 (4.84)***	0.09 (4.68)***	0.07 (3.65)***	0.07 (3.72)***
Having financial liabilities		−0.13 (−1.83)*	−0.14 (−1.87)*	−0.10 (−1.42)	−0.11 (−1.69)*
Years of education			0.03 (1.90)*	0.02 (1.91)*	0.02 (1.77)*
Financial literacy			0.03 (0.39)	−0.08 (−0.93)	−0.05 (−0.62)
Debt literacy			−0.01 (−0.12)	−0.07 (−1.04)	−0.08 (−1.34)
Numeracy			−0.08 (−0.69)	−0.04 (−0.39)	−0.04 (−0.40)
Verbal intelligence			0.004 (0.76)	0.002 (0.43)	0.003 (0.66)
Numerical intelligence			0.0004 (0.11)	0.002 (0.44)	0.001 (0.32)
Figural intelligence			−0.01 (−1.96)*	−0.01 (−2.50)**	−0.01 (−2.11)**
Risk Optimism Index (ROI)				0.14 (5.78)***	0.10 (3.82)***
Risk Tolerance Index (RTI)					0.08 (3.60)***
N	157	157	157	157	157
Adjusted R^2^	0.04	0.17	0.19	0.34	0.39

Right AI activation is included as an independent variable of interest. Coefficients are shown with t−statistics in parentheses.

Significance levels: *p < 0.1, **p < 0.05, ***p < 0.001.

To rule out a lack of significant results due to measurement issues, we additionally tested group differences within each economic variable and found that in concurrence with previous literature^16,17,22^, participants’ household income (two-sample t-test, p < 0.001, active stock traders: 5.9 ± 1.7 SD, non-active stock traders: 4.6 ± 1.6 SD, n = 157) and the years of education (two-sample t-test, p = 0.014, active stock traders: 16.7 ± 2.2 SD, non-active stock traders: 15.6 ± 2.5 SD, n = 157) were significantly different between the two groups.

### Beliefs (risk optimism) and preferences (risk tolerance) explain the association between AI activation and real-life stock trading

**Figure 4:**
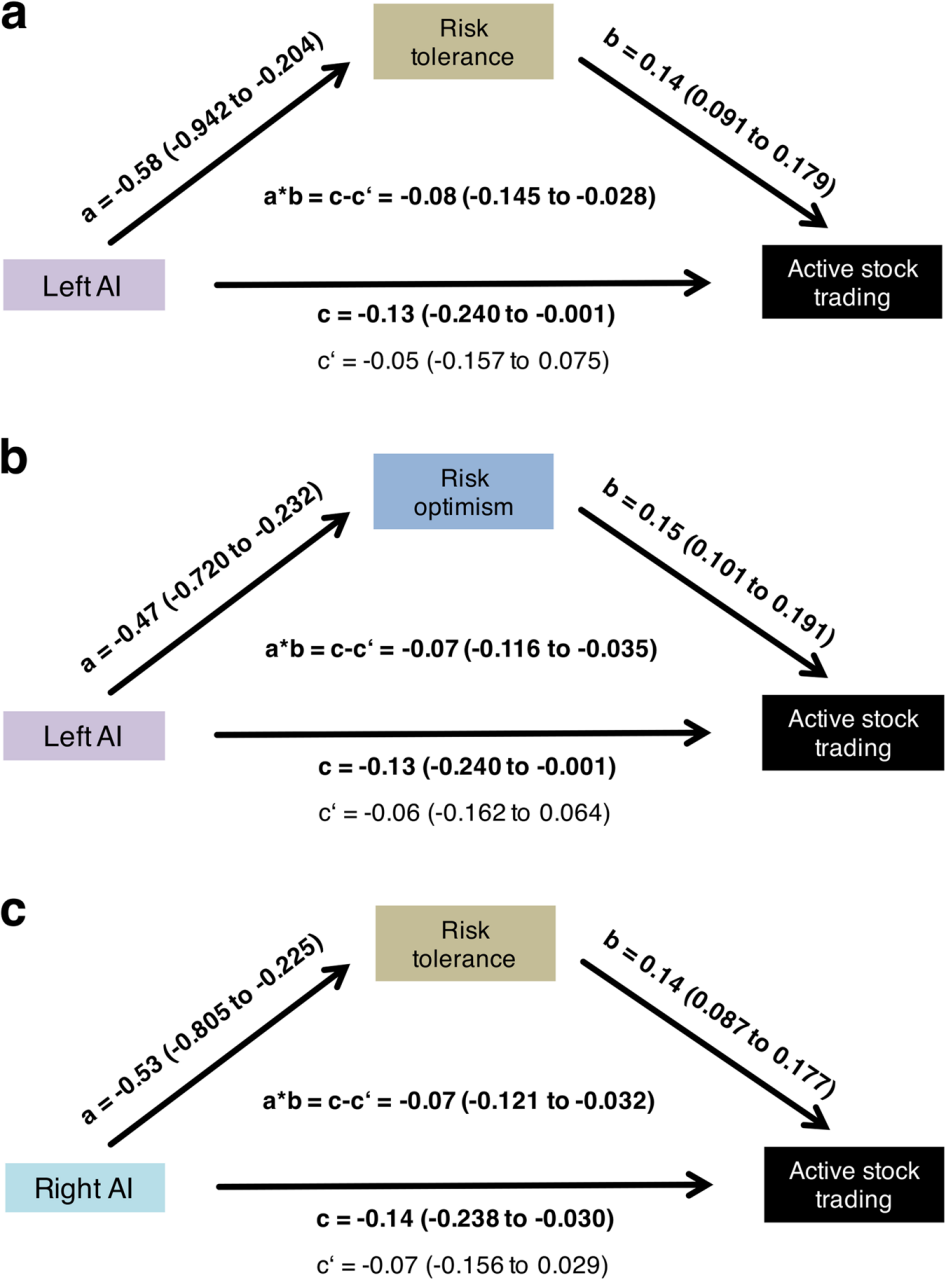
The association between the activation in the anterior insula (AI, independent variable, when choosing between a stock and a bond) and active stock trading (dependent variable) is mediated through risk tolerance and risk optimism (mediator variable, see Supplementary Table S10). The Sobel-Goodman (SG) Mediation test was used with subsequent bootstrapping of the effect (all n = 157, seed set at 10, 10,000 repetitions). Observed coefficients are shown with 95% (bias-corrected and accelerated) confidence intervals in parentheses. An effect is considered significant if the confidence interval does not include the null hypothesis (i.e. zero is not included) and is visualized in bold. (**a**) Mediation of left AI activation and active stock trading through risk tolerance (at least 17.7%). (**b**) Mediation of left AI activation and active stock trading through risk optimism (at least 22.9%). (**c**) Mediation of right AI activation and active stock trading through risk tolerance (at least 25.0%).

Previous studies have found that experimentally-elicited beliefs and preferences about risk taking influence financial choices in laboratory settings^23,24^. Additionally, single measures of beliefs and preferences have been linked to real-life outcomes^35^ and specifically portfolio decisions^36,37^. However, recent evidence suggests that risk preference reflects the structure of a multifaceted psychological trait and should henceforth be studied with a more comprehensive approach^38^. We therefore created two independent, aggregate measures related to beliefs regarding the outcomes of risky choices (risk optimism index (ROI)) and to the willingness to bear risks (risk tolerance index (RTI)). Here, we found that that the association of the AI activity with active stock trading is mediated through both ROI and RTI. For the creation of ROI and RTI, we first classified all self-assessment and behavioural measures into either the risk optimism or the risk tolerance category (see Supplementary Table S5) and then used regression analyses of active stock trading to assess which variables should be used for a subsequent Principal Component Analysis (PCA; see Supplementary Tables S6 and S7). With PCA, we then computed a primary factor for each category, which we labeled as ROI and RTI (see Supplementary Tables S8 and S9). When we added these primary factors to the model that controlled for the previous factors (i.e., financial and cognitive constraints) we found that both ROI and RTI impacted the association between the left AI and real-life stock trading (Table 2) and that RTI influenced the association between the right AI and real-life stock trading (Table 3). We formally tested the association between AI activation and ROI and RTI in a linear regression framework (Table 4), and then conducted a mediation analysis with subsequent bootstrapping of the effects (see Fig. 4 and Supplementary Table S10). Our evidence indicates that the association between AI activation and real-life stock trading is mediated by individuals’ ROI and RTI, rather than by financial or cognitive constraints.

**Table 4:** Linear regression models showing the effects of risk tolerance and risk optimism on activation in the left and right anterior insula.

	Left AI (stock > bond choice, gain domain)			Right AI (stock > bond choice, gain domain)		
Risk Optimism Index (ROI)	−0.10 (−2.83)**		−0.06 (−1.56)	−0.05 (−1.31)		0.01 (0.12)
Risk Tolerance Index (RTI)		−0.11 (−3.23)**	−0.08 (−2.18)**		−0.12 (−3.20)**	−0.12 (−2.90)**
N	157	157	157	157	157	157
Adjusted R^2^	0.04	0.06	0.07	0.005	0.06	0.05

Coefficients are shown with t-statistics in parentheses.

Significance levels: *p < 0.1, **p < 0.05, ***p < 0.001.

### Additional self-reported and behavioural results

We found significant associations between our a priori determined self-reported active stock trading variable and two independent self-reported real-life financial outcome variables. These were the binary variables of having financial investments (Pearson’s Chi-squared = 20.14, p < 0.001, Cramér’s V = 0.32, n = 198) and stock market participation (having a fraction of financial investments invested into stocks and/or mutual funds; Pearson’s Chi-squared = 63.01, p < 0.001, Cramér’s V = 0.56, n = 198). Additionally, we found that the mean self-assessment of financial risk taking was significantly lower in active stock traders compared with non-active stock traders (p < 0.001, active stock traders: 4.63 ± 1.27 SD, non-active stock traders: 2.96 ± 1.05 SD, n = 194). For the self-assessment of financial risk taking, we also found that when considered separately (rather than as part of the RTI), the self-assessment of financial risk taking was significantly correlated with the extracted bilateral AI brain activation (left AI: r = −0.21, p = 0.007; right AI: r = −0.25, p = 0.002, both n = 163). In the investing paradigm, we found that the reaction time for choosing the stock vs. bond did not differ between the two groups (first trial of each block, p = 0.483, active stock traders: 1.89 s ± 0.97 SD, non-active stock traders: 1.93 s ± 0.98 SD, n = 157) and that the reaction time of participants in both groups decreased throughout the experiment (both p < 0.001, active stock traders: r = −0.17, non-active stock traders: r = −0.22, n = 157). Furthermore, participants were asked to estimate the probability of the stock being good in each trial (Fig. 1). This allowed us to obtain the participants’ average stock assessment error (i.e. the difference between objective and subjective estimation), which was negatively correlated with measures of fluid intelligence (see Supplementary Table S11). Additionally, we found a significant correlation between ROI and RTI (see Supplementary Fig. S3).

### Outcomes of a data-driven approach

To address the concern that our hypothesis-driven creation of ROI and RTI might have influenced the results, we performed a purely data-driven approach. To this end, we combined all individually significant variables (see Supplementary Tables S8 and S9) into one PCA and used the primary factor as an index of financial risk seeking and preference (RSPI, see Supplementary Table S12). We then performed the same analysis as previously done with ROI and RTI. For both the left and right AI, the RSPI significantly influenced the association between brain activation and real-life stock trading (see Supplementary Tables S13 and S14). Furthermore, it also mediated this association (see Supplementary Table S10). However, a comparison with the ROI and RTI results revealed that the data-driven approach resulted in a loss of qualitative information due to concealing differences in the mediation of the neural data (left and right AI differences with respect to ROI and RTI). We therefore refrained from using RSPI, and instead decided to use the two hypothesis-driven^14^ indices RTI and ROI.

## Discussion

In our study, we do not only extend the association between brain activation and active stock trading to behaviour in the real world, but additionally identify the mechanisms that underlie this association. We first show that brain activation in the AI during financial decision making under risk is associated with financial risk taking in real life. We then find that this association is not explained by individual differences in financial constraints, education, the understanding of financial matters, or cognitive abilities. However, we find evidence that the association between AI activation and real-life stock trading is explained by comprehensive measures of individuals’ risk tolerance and beliefs about financial risk taking.

We examine the connection between activation in several brain areas (VS, AI, and vmPFC) previously associated with financial risk taking in the laboratory. We find that only one of these brain areas, namely, the AI, is associated with real-life financial risk taking as measured by a self-reported question of directly trading individual stocks. Our results extend previous research by demonstrating that activation in exactly those AI regions that have been associated with risk prediction^6^ and a higher propensity to sell risky assets^1^ in laboratory tasks transfers to financial risk taking in real life. Participants who show a stronger AI signal when choosing a risky over a safe asset in the gain domain are less likely to trade stocks in real life. In conjunction with the previously demonstrated role of the AI in risk aversion^2,4,7,8,11^, as well as its function as a warning signal that differentiates high from low earners in a stock market bubble experiment^1^, our findings indicate that these signals are decisive for behaviour in real life as well. From a clinical perspective, studies with insular-lesioned patients have unveiled an association with atypical financial risk taking behaviour^39,40^. Additionally, insula dysfunction has previously been shown to be part of the pathology of several neurological disorders^41–43^. This suggests that future research could investigate the financial decision-making behaviour of different clinical populations and find out whether atypical financial decision-making of such populations translates to similar behaviour in real life.

Notably, we find an association between real-life financial risk taking behaviour and AI activation in only the gain and not the loss domain. In our investing paradigm (Fig. 1), the motivation in the gain domain is to win money, while in the loss domain it is to avoid losses. In real life, investments in equities are characterized by positive expected returns. It is therefore possible that investing in stocks in real life is more similar to investing in the “stock” that is available during our lab experiment in the gain condition, where that asset promises positive outcomes. The “stock” in the loss condition is not a natural equivalent of real-life equity markets during normal economic conditions, in that during such times investors expect a positive return – not a loss, which is what our experimental condition promises.

In spite of our results being in line with previous studies of risk aversion signals in the AI, the findings could additionally be interpreted the other way around, thus implying that active stock traders who choose the safe (bond) over the risky (stock) option show higher AI activation in comparison to non-active stock traders. Although this interpretation could tentatively be placed in context with previous findings of regret^44^, in our investing paradigm (Fig. 1) the stock outcome is not known at the time of making the choice (the participant does not know whether he made the right or wrong choice) and we therefore abstain from any further interpretations in this context. Even though we do not find evidence that activation in the VS and vmPFC explains real-life stock trading, it is possible that activation in these regions relates to other aspects of financial choices, for example, how individuals respond to new information about investment options^44,45^. Although beyond the scope of this article, it would be interesting to incorporate additional regions of interest from studies that used different approaches to quantify risk and uncertainty^46–48^ and to investigate the exact neural mechanisms underlying stock trading from a computational perspective. Lastly, whereas our design included only male participants, future work should study female participants, as well.

We do not find that financial constraints, education, the understanding of financial matters, or cognitive abilities explain the interaction between active stock trading and AI activation. However, our comprehensive measures of preferences and beliefs about risk taking explain this interaction. Importantly, we included both self-assessments and behavioural data in the creation of these two aggregate measures, because it was previously demonstrated that behavioural and self-report measures of risk taking are weakly correlated, which suggested that they assess specific features of a complex construct^38,49^. The role of risk tolerance and risk optimism as mediators between brain activation and real-life financial behaviour leaves the question of whether the understanding of the mechanisms behind other real-life financial decisions such as stock market participation or portfolio choices in general^21,50^ could likewise be better understood with a combination of self-assessment, behavioural, and especially neuroscientific analysis.

Although economic indicators of real-life stock trading have previously been identified, self-assessment, behavioural, and neuroscientific research on individual investors has been scarce and mostly confined to the laboratory^14^. This is remarkable considering that individual differences in risk preferences have important consequences for many life domains^14,51,52^. Additionally, understanding the characteristics of real-life stock traders is crucial considering the large number of individuals participating in asset markets and their impact on trading volume and prices. In Organisation for Economic Co-operation and Development (OECD) countries, roughly 23% of households hold shares and other equities^53^. In Germany alone, private households directly own stocks with an estimated total net worth of 158 billion Euros^54^. Here, we provide several explanatory variables of stock trading, which may help policy makers assess why certain households participate in equity markets, as well as help providers of financial services to tailor their advice and products to individuals in accordance with these investors’ characteristics. Furthermore, stock market non-participation can imply welfare losses for households^55^ and understanding the underlying mechanisms of this behaviour might therefore help to support individuals in more efficient ways. For example, consumers who are unduly pessimistic about stock investments, and thus have a weak risk optimism as measured by the ROI, could be educated with respect to the historical performance of equity markets worldwide. Also, consumers could be offered products by financial institutions that have appropriate risk-return profiles given these individuals’ risk preferences, as captured by their RTI.

Concurring with recent suggestions regarding the study of individual brain activation differences^56^, we use fMRI data from a large sample and base our analysis on strong prior hypotheses. Brain activation correlates have been previously documented in the context of economic decisions such as consumer choices and monetary donations^57–60^, but not in the context of financial risk taking. This study extends our knowledge of financial risk taking from the laboratory to the real world, which is an important next step in understanding individual heterogeneity in real-life financial decisions and in helping individuals to make better financial choices.

## Methods

### Experimental Design

210 men were invited to the Life & Brain Center at the University Clinic in Bonn (Germany) to participate in an experiment investigating the underpinnings of financial risk taking. Before coming to the appointment, participants underwent screening for the absence of neurologic, psychiatric, and cardiovascular diseases. Other exclusion criteria were ages below 29 and above 50, having a student status, unemployment longer than three months, very bad eyesight (more than ±5 diopters), excessive smoking (more than ten cigarettes per day), as well as large tattoos above the waist. We asked only men to participate in the experiment, because previous studies have shown that the menstrual cycle impacts both financial risk seeking^61^ and reward-related brain activation^62^ in women. The experiment involved no deception and was approved by the ethics committee of the University of Bonn. Each participant gave informed written consent according to the Declaration of Helsinki^63^ and the participants were made aware of the nature and consequences of the study. The experimental session consisted of the following parts: introduction and training, structural magnetic resonance imaging (MRI) with a concurrent behavioural task (stock allocation task, see Supplementary Fig. S4), functional MRI (fMRI) with an investing paradigm (Fig. 1), diffusion tensor imaging (DTI), intelligence tests, a financial questionnaire (Supplementary Document, see Supplementary Information), two personality tests (NEO-FFI^64^ and rRST-Q^65^), and blood collection. Compensation consisted of a basic payment of 20 Euro per hour, with possible addition or subtraction depending on the results of the behavioural and fMRI task. Participants were always paid a minimum of 70 Euros for their average attendance of three and a half hours. The personality, DTI, and blood-related results will be reported in other publications.

After the initial introduction and training session (both the practice and the full fMRI task are deposited in the Supplementary Database, see Supplementary Information), participants were asked to lie in a Siemens Trio 3.0 T scanner (Siemens, Erlangen, Germany) and were accustomed to special response grips (Nordic NeuroLab, Bergen, Norway). The subjects viewed the experimental screen via video goggles (Nordic NeuroLab, Bergen, Norway) that were fixated on a head coil. A system update of the MRI scanner at the end of 2013 led to a mandatory adjustment of the T1 protocols, thus resulting in two ways of structural imaging data collection. Before the scanner update, participants underwent measurements with a standard 8-channel head coil. The scans commenced with a localizer scan that was followed by a structural scan containing T1-weighted images (TR = 1570 ms; TE = 3.42 ms; flip angle = 15). After the scanner update, a standard 12-channel head coil was used and the scans also started with a localizer scan, followed by a structural scan containing T1-weighted images (TR = 1660 ms; TE = 2.75 ms; flip angle = 9). During the T1 measurement, participants completed a behavioural paradigm investigating financial risk taking. This was a stock allocation task^66^ (see Supplementary Fig. S4), which was implemented in Presentation v14 (the paradigm is deposited in the Supplementary Database, see Supplementary Information; Neurobehavioral Systems, Berkeley, California, USA). In the stock allocation task, each subject was asked to make ten independent investment decisions by splitting up a fixed maximum investment amount of 23 Euro to either a risky (stock) or a riskless (bond) asset. Each subject saw two equally likely stock return rates, the bond return rate, and a reminder of the maximum investment amount. These return rates varied in each trial, during which subjects were asked to enter the amount that they would like to invest in the stock, with the remaining funds automatically being invested in the bond. The average amount allocated to the stock was taken as an estimate of individual risk preference, thus meaning that a higher investment represented higher levels of financial risk taking. The return rate was only revealed at the end of the experiment, at which point one trial was randomly selected by the program and integrated in the total compensation.

The investing task (Fig. 1, the paradigm is deposited in the Supplementary Database, see Supplementary Information) was adapted from a previously established behavioural paradigm^25^ to an fMRI setting to measure the blood-oxygenated-level dependent (BOLD) signal during financial investment decisions. It was implemented in Presentation v14 (Neurobehavioral Systems, Berkeley, California, USA). While subjects completed the paradigm, T2*-weighted echoplanar images (EPIs) were collected using a standard 8-channel (before the scanner update) or 12-channel (after the scanner update) head coil. The scanning parameters were similar both before and after the scanner update (TR = 2500 ms; TE = 30 ms; flip angle = 90; 37 3 mm slices in ascending order; field of view = 192 mm; approx. 840 volumes) except for a change in voxel size (before the scanner update: 3 × 3 × 3 mm; after the scanner update = 2 × 3 × 3 mm). Each participant started with an initial endowment of 25 Euros and had to choose between a risky (stock) and a non-risky (bond) financial option in 96 total trials. Before each set of six trials, which made up one of the 16 blocks, it was shown to the subject for 2 s whether he was in the loss or the gain condition. Each participant completed 16 blocks (eight gain and eight loss), shown in random order. An initial screen of a block was followed by a jittered interstimulus interval (ISI, 1 to 3 s). The participants then had maximally 3 s to decide between a stock and a bond. Two risk levels seen via possible stock outcomes were implemented and remained consistent throughout each block: high risk (0 vs. 12 Euro or −12 vs. 0 Euro) and low risk (2 vs. 10 or −2 vs. −10 Euro). Each subject completed each of the four conditions (gain − high risk; gain − low risk; loss - high risk; loss - low risk) in a pseudo-randomized fashion four times. The bond always remained the same (6 or −6 Euros) in each condition. The chosen option was highlighted with a green frame for 0.75 s. After another jittered ISI of 3.5 to 7 s and independent of the choice, the outcome of the stock was shown for 3 s. Each block was randomly assigned to contain either a good or a bad stock. Due to the thorough instructions and practice trials given before entering the MRI scanner, subjects knew that a good stock had a higher outcome probability of 70% and a lower outcome probability of 30%. A bad stock had the opposite probabilities. An objective Bayesian probability of the stock being either good or bad was estimated using the previous outcomes within the same block. This estimate was not presented to the participants. The subjects then had up to 3 s to enter a subjective probability estimate of the stock being good each time after having seen the stock outcome. Participants received an incentive of 10 Euro cents for each answer within 5% of the objective estimation. This amount was then added to the final balance. At the end of each trial, the subjects saw the updated balance for 3 s. A behavioural variable from the investing paradigm that was included in the analysis was the ratio of stock to bond choices, but only in the first trial of each block (used as a measure of behavioural risk taking). This was done to avoid any bias by the learned stocks’ outcome probabilities. Two additional behavioural variables were obtained via the investing paradigm. The average absolute value of the difference between the objective and the subjective estimate of the stock being a good stock was taken as a measure of risk learning and was termed stock estimation error. Its non-absolute variant was taken as a behavioural measure of risk optimism.

After the scanning session, participants completed three subscales of the Intelligence-Structure-Test (IST) 2000R: verbal, numerical, and figural intelligence^34^. These subscales included questions concerning analogies (verbal intelligence), numerical series (numerical intelligence), and matrices (figural intelligence). The subscale scores were normalized to the respective age groups (26 to 30, 31 to 40, and 41 and older) and resulted in three variables of interest: verbal, numerical, and figural intelligence (Table 1).

After the intelligence test, participants were asked to fill in a questionnaire consisting of demographic, risk preference, as well as financial knowledge questions (Supplementary Document, see Supplementary Information). These questions were mainly taken from two sources: the German Socio-Economic Panel (SOEP) study developed by the German Institute for Economic Research (Deutsches Institut für Wirtschaftsforschung (DIW))^67^, as well as from a questionnaire created by the Munich Center for the Economics of Aging (MEA). The MEA questions aimed at assessing financial knowledge and real-life financial decisions. These questions investigated debt literacy^68^, financial literacy^69^, and numeracy^70^ and were previously used in other surveys such as the Survey of Health, Ageing and Retirement in Europe (SHARE). Additional demographic financial questions were specifically created for this study. An example of this was our binary dependent variable asking about active stock trading (“Do you trade stocks yourself?”, question 21 in the Supplementary Document, see Supplementary Information). In total, 9 variables from the handout were chosen for our analysis (Table 1). Most of these variables were represented by one question, except for years of education, financial literacy, debt literacy, and numeracy. The years of education were the sum of the following fixed years in the German school system: main school/Hauptschule (9 years), secondary school/Mittlere Reife (10 years), ten-class general educational polytechnic secondary school/Polytechnische Oberschule (10 years), university of applied sciences entrance qualification/Fachhochschulreife (12 years), high-school diploma/Abitur (13 years); the higher education years were set as follows: none (0 years), university degree/Hochschulabschluss (5 years (average diploma time)), Apprenticeship/Lehre & Meisterschule (1.5 years), training for civil servants/Beamtenausbildung (2 Jahre), college/Fachhochschule (4 years (average diploma time)), others (1 year). Regarding financial literacy (questions 39, 40, and 41 in the Supplementary Document, see Supplementary Information), debt literacy (questions 42 and 43), and numeracy (questions 45, 46, and 47), the amount of correct answers was calculated to represent each variable. The numbers of the relevant questions taken from the handout (Supplementary Document, see Supplementary Information) are listed in Table 1. After completing the handout, participants completed two personality questionnaires (NEO-FFI^64^ and rRST-Q^65^), blood was collected, and the participants got to know their final payout, which was delivered to the participants via money transfer.

### Exclusions

Out of the invited 210 participants, twelve participants had to be fully excluded due to claustrophobia, neurological diseases or psychological disorders, and failing to return the consent form. Furthermore, 33 participants were excluded from the analysis that included fMRI data due to technical issues during data acquisition and excessive movements of participants during the fMRI task (>2.5°, >5 mm). The remaining 165 participants were on average 38.9 ± 6.7 SD years old. Of these 165 participants, 43 were measured before a mandatory scanner update and 122 afterwards.

### Statistical Analysis

#### Functional magnetic resonance imaging (fMRI) analysis

Functional magnetic resonance imaging (fMRI) datasets from 165 participants were used for the fMRI second-level analysis. Preprocessing of the functional images was done using Statistical Parametric Mapping 12 (SPM12, Wellcome Department of Imaging Neuroscience, London, UK) implemented in MATLAB R2014 (MathWorks, Natick, Massachusetts, USA). Preprocessing included realignment, slice-time correction, spatial normalization to the Montreal Neurological Institute (MNI) space using the anatomical T1 image of each participant, and a final smoothing step using a Gaussian kernel with full-width at half-maximum (FWHM) of 8 mm.

One GLM was specifically designed to estimate brain activation during reward prediction error (RPE) processing (GLM described in Supplementary Table S1) and another GLM was used to estimate brain activation during the choice and feedback phase (see Supplementary Table S2, this script can be found in the Supplementary Database, see Supplementary Information). Both models included the canonical hemodynamic response function (HRF) implemented in SPM12. They also included a high-pass filter of 128 Hz as well as a correction for autocorrelations. The onset regressors of the first GLM were the onset of the choice screen, stock payoff feedback, stock estimation, and balance feedback, which were all further split into trials when the subject chose the stock and trials when the subject chose the bond (see Supplementary Table S1). Additionally, the stock payoff feedback onset had three parametric modulators, consisting of the RPE, the reward prediction (RP), and the trial payoff (see Supplementary Table S1). All parametric modulators were orthogonalised in ascending order. The RP was calculated as the objective probability of the stock being good. The RPE was calculated as the difference between the updated objective probability of the stock being good at the time of the newly presented payoff feedback and the objective probability of the stock being good at the time before the new payoff feedback was presented. The activation that correlated positively with the RPE at the onset of the payoff feedback after having chosen the stock was used to assess the reliability of the paradigm (Fig. 1) in relation to previous literature, which consistently found RPE-related activation in the VS and the vmPFC^2,9,10,15,26–30^.

The onset regressors used in the second GLM were the onset of choice screen, stock payoff feedback, stock estimation, and balance feedback (see Supplementary Table S2). Each of these regressors were divided into the gain and loss domain, as well as having chosen the stock or the bond. The stock payoff feedback regressors were additionally split into a good or a bad outcome (see Supplementary Table S2). The onset of the choice screen was given the duration of the time until button press and the other regressors were modeled as stick functions. The only parametric modulator was the trial payoff, which was used during the payoff feedback. Following the estimation of this GLM, 14 contrasts of interest were defined and analyzed on the group level (a sample script of this GLM and all contrast results for the group-level analysis can be found in the Supplementary Database, see Supplementary Information).

#### Weighted beta value extraction and their association with active stock trading

Next, five regions of interest were used to extract weighted beta estimates from the choice and stock payoff feedback contrasts (Fig. 2b, all regions of interest masks are deposited in the Supplementary Database, see Supplementary Information). The first region of interest was the vmPFC (MNI coordinates at around 0, 46, −8), obtained from a meta-analysis concerning the valuation system (see vmPFC mask in Fig. 9 of ^9^). The second and third regions of interest were 6 mm radius spheres in the bilateral VS (MNI coordinates: ±12, 8, −8) and the right anterior insula (AI, MNI coordinates: 36, 24, 2), obtained from the authors of a recent neuroeconomic study investigating reward and loss activation during a stock market experiment^1^. The fourth region of interest was made using the MARSeille Boîte À Région d’Intérêt (MarsBaR) toolbox implemented in Matlab and creating a 6 mm radius sphere at the location of the left AI (MNI coordinates: −32, 25, 3). This location was taken from Table S3 of a previous study assessing risk^6^ and was the same “risk prediction signal” table that was previously used for the creation of the right AI in a study that investigated trading behaviour under risk in a stock market experiment^1^. Due to the fact that the coordinates were in Talairach space (−31, 22, 7.7), we contacted the authors of the previous neuroeconomic publication^1^ in order to use the same MNI to Talairach converter^71^. As a control variable and fifth region of interest, we created a 6 mm radius sphere in the right fusiform face area (FFA, MNI coordinates: 40, −50, −18) as described in a meta-analysis of 105 functional MRI studies assessing emotional face processing^31^. Using these regions of interests, weighted beta estimates were extracted for all contrasts (see Supplementary Table S4), except for the FFA mask, which was only used to extract weighted beta estimates for the first contrast. Kernel density plots were then made for each weighted beta estimate (see Fig. 3 and Supplementary Figs S1 and S2).

The analysis was done using STATA version 13 (Stata-Corp LP, College Station, Texas, USA). The data set and script can be found in the Supplementary Database, see Supplementary Information. Before continuing with the analysis, it was established that 157 complete datasets could be used to study active stock trading. At first, two sample t-tests (uncorrected) were performed to see which weighted beta estimates were significantly associated with the self-reported measures of real-life stock trading (see Supplementary Table S4). Because only the left and right AI revealed significant activation for the contrast stock choice vs. bond choice in the gain domain, we focused on the bilateral AI for the subsequent analysis.

#### Investigation of the possible mechanisms behind the association between AI activation and real-life stock trading

We first selected variables that were previously related to stock market participation and that could influence the association between the AI activation and real-life stock trading (Tables 1 to 3; distribution plots are shown in Fig. 3). These included variables that assessed financial constraints^16,17^, education^16,17,22^, the understanding of financial matters^20,21^, and cognitive abilities^18,19^ (Table 1). Next, we tested their influence on the AI and real-life stock trading association using multiple regression analysis (Tables 2 and 3).

In line with previous financial risk taking literature that used the concept of beliefs and preferences^14^, we then grouped all risk-related behavioural and self-assessment measures into categories of risk tolerance and optimism. The risk optimism category included variables that related to beliefs regarding the outcomes of risky choices and the risk tolerance category included variables relating to the willingness to bear risks (see Supplementary Table S5). Guided by economic theory, logistic regressions using active stock trading as the dependent variable (DV) were calculated for each of the variables individually (see Supplementary Tables S6 and S7). The significantly associated variables from the risk optimism and risk tolerance categories were then used for principal component analysis (PCA, see Supplementary Tables S8 and S9). This PCA approach was already successfully used in risk taking research before, specifically to determine whether a single principal component was able to determine risk taking in several contexts^36^. The primary components found in the two PCAs (see Supplementary Tables S8 and S9) were labeled risk optimism index (ROI) and risk tolerance index (RTI). These indices of preferences and beliefs about risk taking (their distribution is shown in Fig. 3) were then added to the previous regression models to test their influence on the association of the AI activation and real-life stock trading (Tables 2 and 3). We ex-ante expected that AI activation, ROI and RTI would have independent contributions to real-life stock trading. However, we found that the connection between AI and real-life stock trading became insignificant once ROI and RTI were included as possible explanatory variables of active stock trading (Tables 2 and 3). As a result, we conducted ex-post regression analyses with the left and right AI activation as the dependent variables and either ROI or RTI, or both indices, as the independent variables (Table 4). To then formally test the mechanism behind the association of AI activation and real-life stock trading, we performed a mediation analysis using the Sobel-Goodman (SG) mediation test with subsequent bootstrapping of the effect, in which we used the ROI and RTI as mediator variables (see Fig. 4 and Supplementary Table S10). A mediation was considered significant if the indirect effect (a*b), but not the direct effect (c) were significant (see Fig. 4 and Supplementary Table S10).

### Data and code availability

The relevant data, code, and materials are deposited in the Harvard Dataverse. They can be accessed via this link, which contains a “.zip” file with the questionnaire (Supplementary Document, see Supplementary Information), as well as the Matlab scripts and regions of interests used in the study. Additionally, all the group-level SPM “.mat” files described in the fMRI contrast overview table (see Supplementary Tables S2 and S3) are included in the “.zip” file. Finally, both the behavioural experiments (stock allocation task and investing paradigm) are included, as well as the final data set and scripts used for the analysis (programmed in Stata v13.1).

## Electronic supplementary material

Supplementary Information

## References

[1] Smith A, Lohrenz T, King J, Montague PR, Camerer CF (2014). Irrational exuberance and neural crash warning signals during endogenous experimental market bubbles. PNAS.

[2] Kuhnen CM, Knutson B (2005). The neural basis of financial risk-taking. Neuron.

[3] De Martino B, O’Doherty JP, Ray D, Bossaerts P, Camerer C (2013). In the mind of the market: Theory of mind biases value computation during financial bubbles. Neuron.

[4] Rudorf S, Preuschoff K, Weber B (2012). Neural correlates of anticipation risk reflect risk preferences. J. Neurosci..

[5] Preuschoff K, Bossaerts P, Quartz SR (2006). Neural differentiation of expected reward and risk in human subcortical structures. Neuron.

[6] Preuschoff K, Quartz SR, Bossaerts P (2008). Human insula activation reflects risk prediction errors as well as risk. J. Neurosci..

[7] Mohr PNC, Biele G, Heekeren HR (2010). Neural processing of risk. J. Neurosci..

[8] Singer T, Critchley HD, Preuschoff K (2009). A common role of insula in feelings, empathy and uncertainty. Trends Cogn. Sci..

[9] Bartra O, McGuire JT, Kable JW (2013). The valuation system: A coordinate-based meta-analysis of BOLD fMRI experiments examining neural correlates of subjective value. Neuroimage.

[10] Clithero JA, Rangel A (2014). Informatic parcellation of the network involved in the computation of subjective value. SCAN.

[11] Leong JK, Pestilli F, Wu CC, Samanez-Larkin GR, Knutson B (2016). White-matter tract connecting anterior insula to nucleus accumbens correlates with reduced preference for positively skewed gambles. Neuron.

[12] Knutson B, Huettel SA (2015). The risk matrix. Curr. Opin. Behav. Sci..

[13] Falk A, Heckman JJ (2009). Lab experiments are a major source of knowledge in the social sciences. Science.

[14] Frydman C, Camerer CF (2016). The psychology and neuroscience of financial decision making. Trends Cogn. Sci..

[15] Häusler AN, Becker B, Bartling M, Weber B (2015). Goal or gold: Overlapping reward processes in soccer players upon scoring and winning money. PLoS One.

[16] Haliassos M, Bertaut CC (1995). Why do so few hold stocks?. Econ. J..

[17] Guiso L, Haliassos M, Japelli T (2003). Household stockholding in Europe: Where do we stand and where do we go?. Econ. Policy.

[18] Benjamin DJ, Brown SA, Shapiro JM (2013). Who is ‘Behavioral’? Cognitive ability and anomalous preferences. J. Eur. Econ. Assoc..

[19] Grinblatt M, Keloharju M, Linnainmaa J (2011). IQ and stock market participation. J. Finance.

[20] van Rooij M, Lusardi A, Alessie R (2011). Financial literacy and stock market participation. J. financ. econ..

[21] Yoong J (2010). Financial illiteracy and stock market participation: Evidence from the RAND American Life Panel. Pension Res. Counc. Work. Pap..

[22] Cole, S., Paulson, A. & Shastry, G. K. Smart money: The effect of education on financial behavior. *Harvard Bus. Sch. Financ. Work. Pap*. 09–071 (2012).

[23] Kuhnen CM, Knutson B (2011). The influence of affect on beliefs, preferences, and financial decisions. J. Financ. Quant. Anal..

[24] Bassi A, Colacito R, Fulghieri P (2013). ’Osole mio: An experimental analysis of weather and risk attitudes in financial decisions. Rev. Financ. Stud..

[25] Kuhnen CM (2015). Asymmetric learning from financial information. J. Finance.

[26] Schultz W, Dayan P, Montague PR (1997). A neural substrate of prediction and reward. Science.

[27] Knutson B, Westdorp A, Kaiser E, Hommer D (2000). FMRI visualization of brain activity during a monetary incentive delay task. Neuroimage.

[28] Fliessbach K (2010). Retest reliability of reward-related BOLD signals. Neuroimage.

[29] Samanez-Larkin GR, Knutson B (2015). Decision making in the ageing brain: Changes in affective and motivational circuits. Nat. Rev. Neurosci..

[30] Häusler AN, Artigas SO, Trautner P, Weber B (2016). Gain- and loss-related brain activation are associated with information search differences in risky gambles: An fMRI and eye-tracking study. eNeuro.

[31] Fusar-Poli P (2009). Functional atlas of emotional faces processing: A voxel-based meta-analysis of 105 functional magnetic resonance imaging studies. J. Psychiatry Neurosci..

[32] Nusslock R, Miller GE (2016). Early-life adversity and physical and emotional health across the lifespan: A neuroimmune network hypothesis. Biol. Psychiatry.

[33] Hanson JL (2015). Cumulative stress in childhood is associated with blunted reward-related brain activity in adulthood. SCAN.

[34] Liepmann, D., Beauducel, A., Brocke, B. & Amthauer, R. *Intelligenz-Struktur-Test2000 R*. (Hogrefe, 2007).

[35] Sutter M, Kocher MG, Glätzle-Rützler D, Trautmann ST (2013). Impatience and uncertainty: Experimental decisions predict adolescents’ field behavior. Am. Econ. Rev..

[36] Dohmen T (2011). Individual risk attitudes: Measurement, determinants, and behavioral consequences. J. Eur. Econ. Assoc..

[37] Barsky RB, Juster FT, Kimball MS, Shapiro MD (1997). Preference parameters and behavioral heterogeneity: An experimental approach in the health and retirement study. Q. J. Econ..

[38] Frey R, Pedroni A, Mata R, Rieskamp J, Hertwig R (2017). Risk preference shares the psychometric structure of major psychological traits. Sci. Adv..

[39] Clark L (2008). Differential effects of insular and ventromedial prefrontal cortex lesions on risky decision-making. Brain.

[40] Weller JA, Levin IP, Shiv B, Bechara A (2009). The effects of insula damage on decision-making for risky gains and losses. Soc. Neurosci..

[41] Namkung H, Kim S-H, Sawa A (2017). The insula: An underestimated brain area in clinical neuroscience, psychiatry, and neurology. Trends Neurosci..

[42] Yao Z, Wang L, Lu Q, Liu H, Teng G (2009). Regional homogeneity in depression and its relationship with separate depressive symptom clusters: A resting-state fMRI study. J. Affect. Disord..

[43] Liu Z (2010). Decreased regional homogeneity in insula and cerebellum: A resting-state fMRI study in patients with major depression and subjects at high risk for major depression. Psychiatry Res. - Neuroimaging.

[44] Frydman CD, Camerer CF (2015). Neural evidence of regret and its implications for investor behavior. SSRN Electron. J..

[45] Kuhnen, C. M., Rudorf, S. & Weber, B. The effect of prior choices on expectations and subsequent portfolio decisions. *National Bureau of Economic Research* (2017).

[46] Huettel SA (2005). Decisions under Uncertainty: Probabilistic Context Influences Activation of Prefrontal and Parietal Cortices. J. Neurosci..

[47] Huettel SA, Stowe CJ, Gordon EM, Warner BT, Platt ML (2006). Neural signatures of economic preferences for risk and ambiguity. Neuron.

[48] Christopoulos GI, Tobler PN, Bossaerts P, Dolan RJ, Schultz W (2009). Neural correlates of value, risk, and risk aversion contributing to decision making under risk. J. Neurosci..

[49] Mamerow L, Frey R, Mata R (2016). Risk taking across the life span: A comparison of self-report and behavioral measures of risk taking. Psychol. Aging.

[50] Grinblatt M, Ikäheimo S, Keloharju M, Knüpfer S (2016). IQ and mutual fund choice. Manage. Sci..

[51] Anderson LR, Mellor JM (2008). Predicting health behaviors with an experimental measure of risk preference. J. Health Econ..

[52] Bellante D, Link AN (1981). Are public sector workers more risk averse than private sector workers?. ILR Rev..

[53] OECD. National Accounts of OECD Countries. *OECD Publ*. (2017).

[54] Deutsche Bundesbank (German National Bank). Panel on household finances 2016. March, 61–86 (2016).

[55] Calvet LE, Campbell JY, Sodini P (2007). Down or out: Assessing the welfare costs of household investment mistakes. J. Polit. Econ..

[56] Dubois J, Adolphs R (2016). Building a science of individual differences from fMRI. Trends Cogn. Sci..

[57] Tusche A, Bode S, Haynes J-D (2010). Neural responses to unattended products predict later consumer choices. J. Neurosci..

[58] Ma Y, Wang C, Han S (2011). Neural responses to perceived pain in others predict real-life monetary donations in different socioeconomic contexts. Neuroimage.

[59] Levy I, Lazzaro SC, Rutledge RB, Glimcher PW (2011). Choice from non-choice: Predicting consumer preferences from Blood Oxygenation Level-Dependent signals obtained during passive viewing. J. Neurosci..

[60] Berkman ET, Falk EB (2013). Beyond brain mapping: using neural measures to predict real-world outcomes. Curr. Dir. Psychol. Sci..

[61] Lazzaro SC, Rutledge RB, Burghart DR, Glimcher PW (2016). The impact of menstrual cycle phase on economic choice and rationality. PLoS One.

[62] Dreher J-C (2007). Menstrual cycle phase modulates reward-related neural function in women. PNAS.

[63] World Medical Association. (2004). World Medical Association Declaration of Helsinki: ethical principles for medical research involving human subjects. Int. J. Bioeth..

[64] Costa, P. T. & McCrae, R. R. *Revised NEO Personality Inventory (NEO PI-R) and NEO Five-Factor Inventory (NEO-FFI): Professional manual*. (Psychological Assessment Resources, 1992).

[65] Reuter M, Cooper AJ, Smillie LD, Markett S, Montag C (2015). A new measure for the revised reinforcement sensitivity theory: Psychometric criteria and genetic validation. Front. Syst. Neurosci..

[66] Kuhnen CM, Chiao JY (2009). Genetic determinants of financial risk taking. PLoS One.

[67] Wagner, G. G., Frick, J. R. & Schupp, J. The German Socio-Economic Panel study (SOEP) - scope, evolution and enhancements. *SOEPpapers Multidiscip*. *Panel Data Res*. 1–32 (2007).

[68] Lusardi, A. & Tufano, P. Debt literacy, financial ecperiences and overindebtedndess. *NBER Work. Pap. Ser. 14808* (2009).

[69] Mitchell, O. S. & Lusardi, A. *Financial literacy: Implications for retirement security and the financial marketplace*. (Oxford University Press, 2011).

[70] Christelis, D., Jappelli, T. & Padula, M. Cognitive abilities and portfolio choice. *CSEF Work. Pap. 157* (2006).

[71] Lancaster JL (2007). Bias between MNI and talairach coordinates analyzed using the ICBM-152 brain template. Hum. Brain Mapp..

