# Supplementary material for: Preferences and beliefs about financial risk taking mediate the association between anterior insula activation and self-reported real-life stock trading
Source: Sci Rep. 2018 Jul 25;8:11207. doi: 10.1038/s41598-018-29670-6 (PMC6060130; doi:10.1038/s41598-018-29670-6)
Supplement: Supplementary file 1 — Supplementary Information [file 41598_2018_29670_MOESM1_ESM.pdf]

Supplementary Information

Title

Preferences and beliefs about financial risk taking mediate the association between anterior insula activation and self-reported real-life stock trading

Authors

Alexander N. Häusler*, Camelia M. Kuhnen, Sarah Rudorf, Bernd Weber

Supplementary Database

The data is deposited in the Harvard Dataverse and can be accessed via this link. It contains a ".zip" file with the questionnaire (Supplementary Document), as well as the Matlab scripts and regions of interests used in the study. Additionally, all the group-level SPM ".mat" files described in the fMRI contrast overview table (see Supplementary Tables S2 and S3) are included in the ".zip" file. Finally, both the behavioural experiments (investing paradigm and stock allocation task) are included, as well as the final data set and scripts used for the analysis (programmed in Stata v13.1).

Supplementary Document

This document is the questionnaire that was used to obtain the demographic, risk preference, as well as financial knowledge variables. It is included in the Supplementary Database ".zip" file, provided via this link.

Supplementary References

1. Hayes, A. F. Miscellaneous topics in mediation analysis. in *Introduction to Mediation, Moderation, and Conditional Process Analysis: A Regression-Based Approach* 165–207 (Guilford Press, 2013).

Supplementary Figures

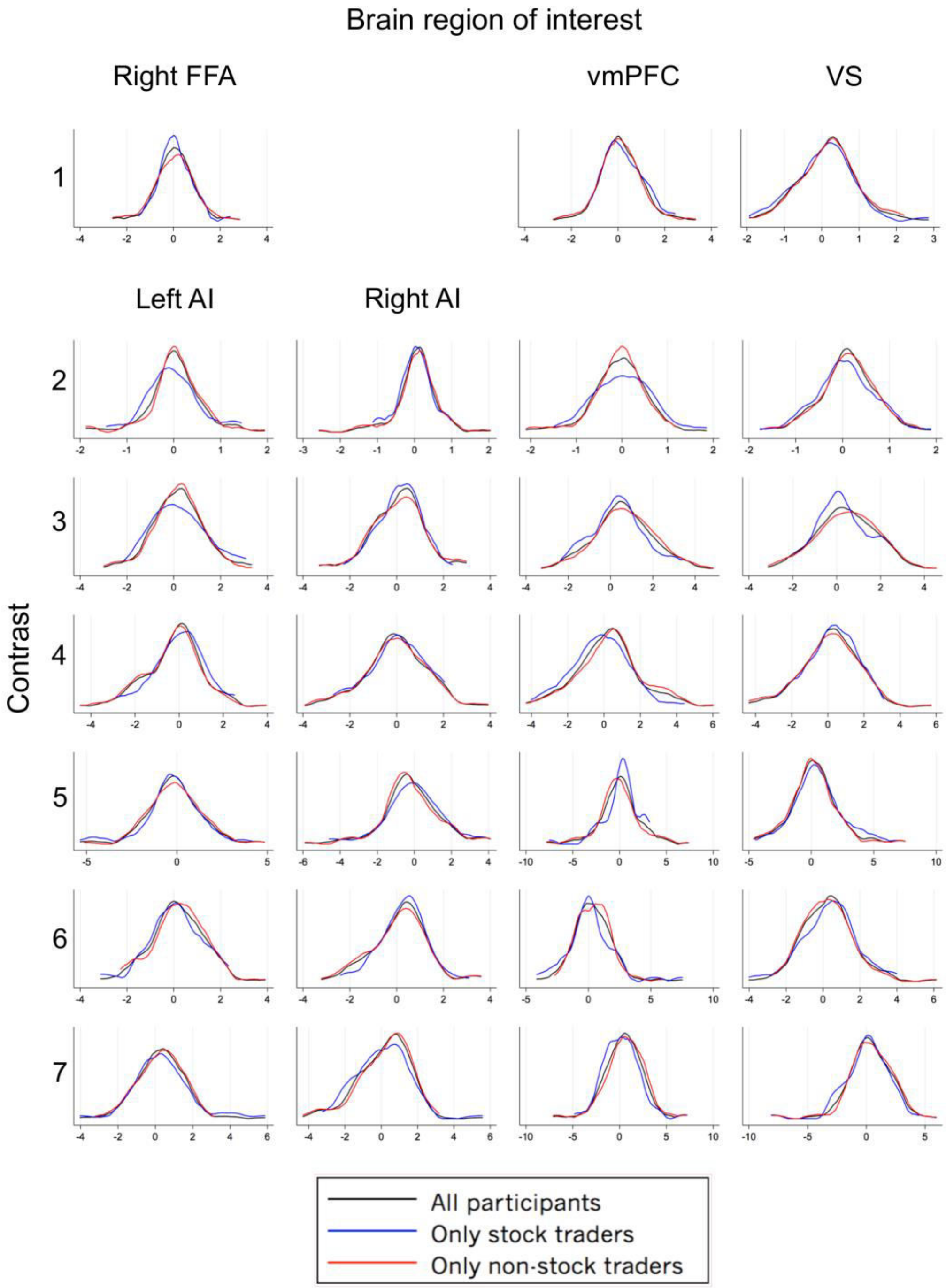

Supplementary Figure S1. Distribution plots of the extracted weighted beta estimates that were not found to be significantly associated with active stock trading (Supplementary Table S4). Here: Contrasts 1 to 7. Each contrast number refers to the contrast number shown in Supplementary Table S4, i.e. contrast 1 = Stock vs. Bond choice (gain domain). Please note that the right FFA was only used for the first contrast as a control variable. Abbreviations: AI: anterior insula; FFA: fusiform face area; vmPFC: ventromedial prefrontal cortex; VS: ventral striatum.

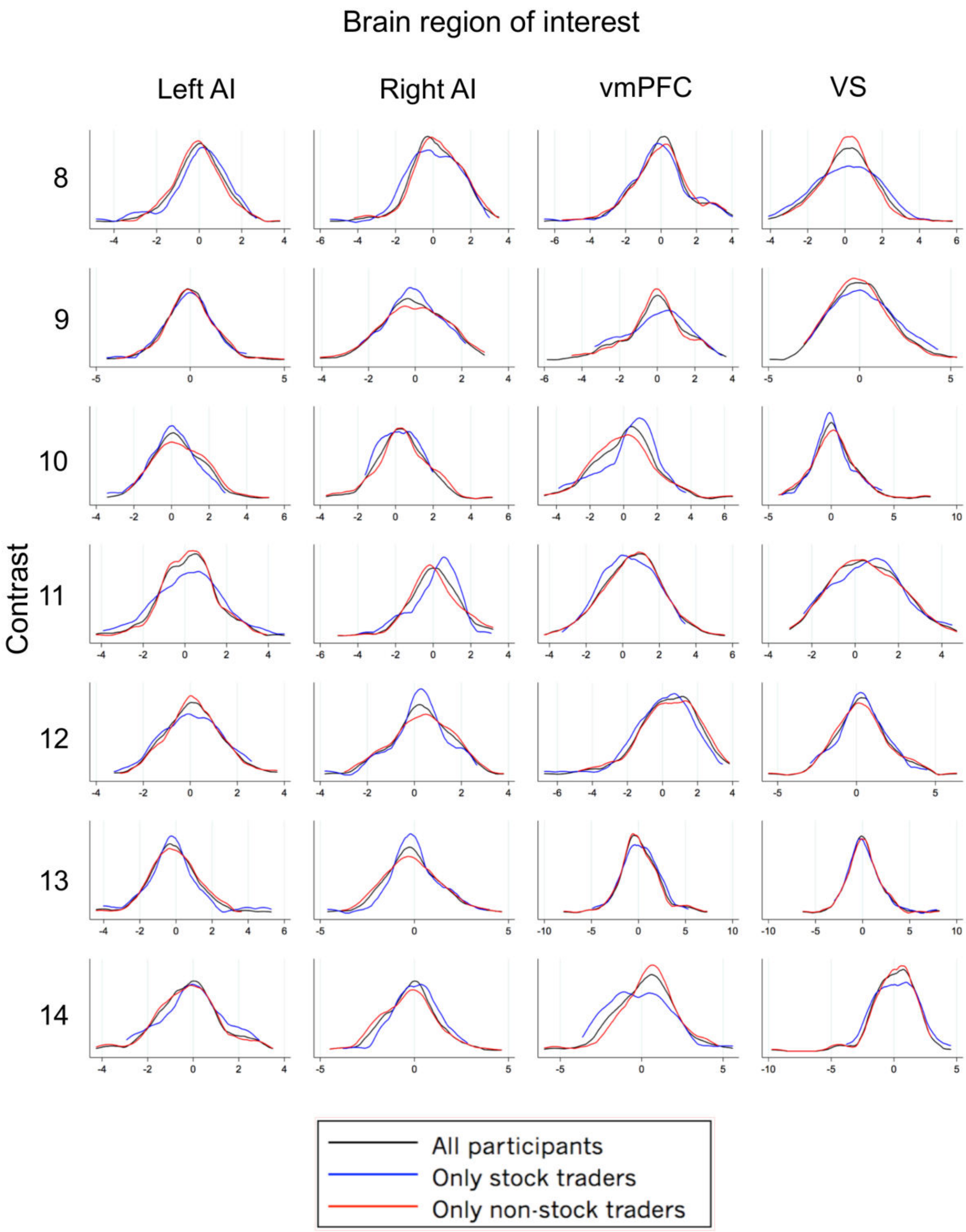

Supplementary Figure S2. Distribution plots of the extracted weighted beta estimates that were not found to be significantly associated with active stock trading (Supplementary Table S4). Here: Contrasts 8 to 14. Each contrast number refers to the contrast number shown in Supplementary Table S4, i.e. contrast 11 = Good feedback after stock choice vs. Bad feedback after bond choice (gain domain). Abbreviations: AI: anterior insula; vmPFC: ventromedial prefrontal cortex; VS: ventral striatum.

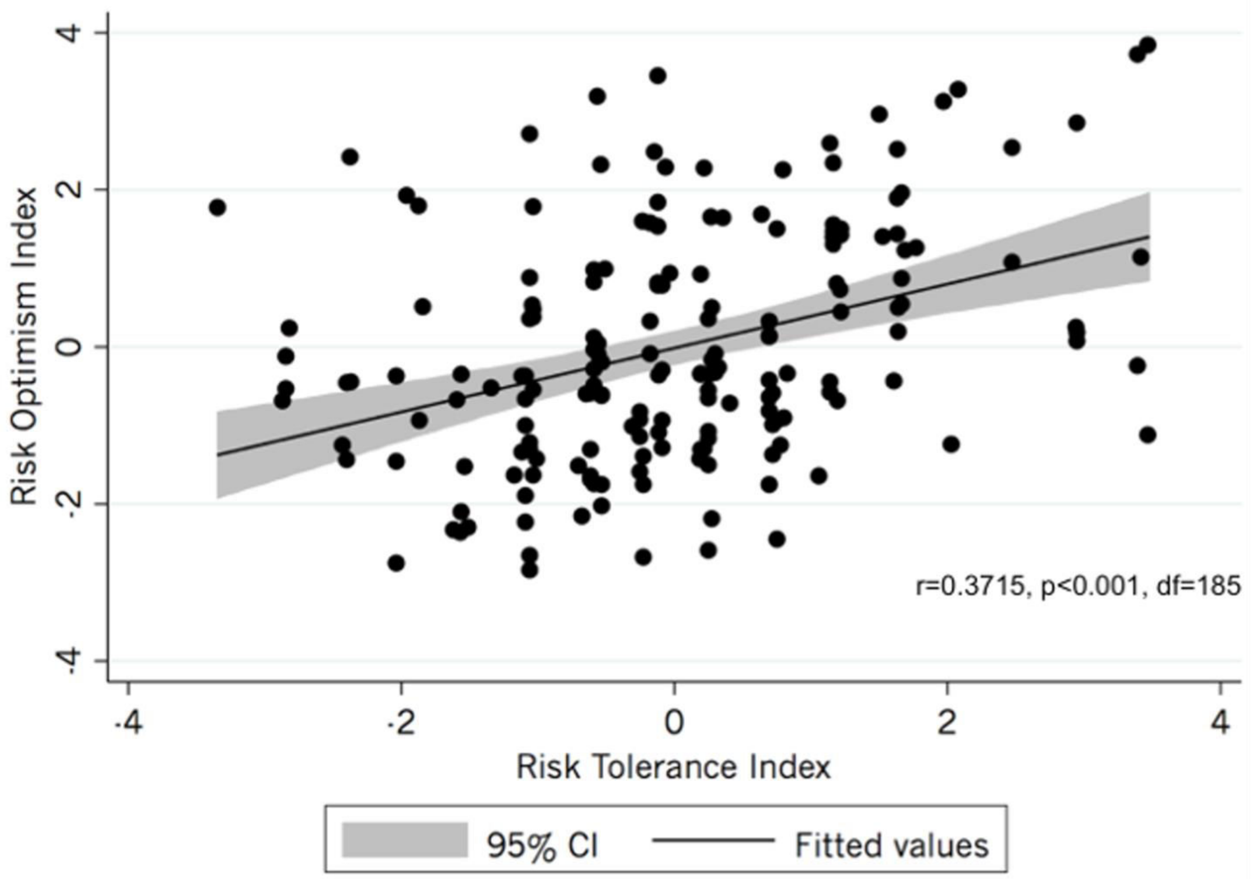

Supplementary Figure S3. Correlation of risk tolerance and risk optimism. The Risk Tolerance Index (RTI) and the Risk Optimism Index (ROI) were the first components of the principal component analyses (Supplementary Tables S8 and S9) using variables that significantly explained parts of the variance of active stock trading (Supplementary Tables S6 and S7).

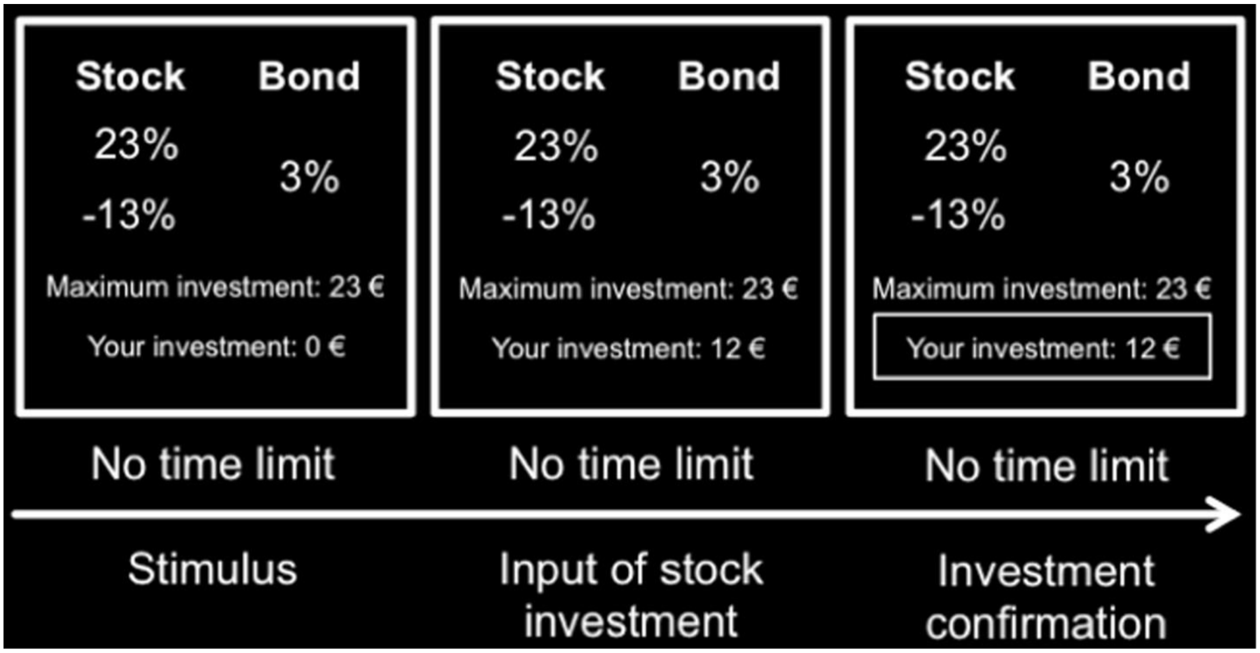

Supplementary Figure S4. Timeline of the behavioural stock allocation task. Each subject was asked to make ten independent financial investment decisions by splitting up 23€ into a risky (stock) and a riskless (bond) option. The average amount of money invested into the stock was taken as a measure of financial risk preference.

Supplementary Tables

Supplementary Table S1. General linear model designed for assessing reward prediction error activation in the investing task.

Regressor number and onset	Parametric modulation	Duration
1. Stock choice	n.a.	Stick function
2. Bond choice	n.a.	Stick function
3. & 4. & 5. & 6. Payoff feedback after stock choice	RPE, RP, and trial payoff	Stick function
7. & 8. & 9. & 10. Payoff feedback after bond choice	RPE, RP, and trial payoff	Stick function
11. Stock estimation after stock choice	n.a.	Stick function
12. Stock estimation after bond choice	n.a.	Stick function
13. Current payoff status after stock choice	n.a.	Stick function
14. Current payoff status after bond choice	n.a.	Stick function
15. - 20. Six movement regressors	n.a.	n.a.
21. Constant	n.a.	n.a.

Abbreviations: n.a. = not applicable; RPE = Reward Prediction Error (calculated as the difference between the updated objective probability of the stock being good at the time of the newly presented payoff feedback and the objective probability of the stock being good at the time before the new payoff feedback was presented), RP = Reward Prediction (calculated as the objective probability of the stock being good).

Supplementary Table S2. General linear model and contrast specification designed for assessing risk-related brain activation in the investing task.

Regressor number and onset	Parametric modulation	Duration
1. Stock choice in gain domain	n.a.	Time until button press
2. Bond choice in gain domain	n.a.	Time until button press
3. Stock choice in loss domain	n.a.	Time until button press
4. Bond choice in loss domain	n.a.	Time until button press
5. & 6. Good payoff feedback after stock choice in gain domain	Trial payoff	Stick function
7. & 8. Bad payoff feedback after stock choice in gain domain	Trial payoff	Stick function
9. & 10. Good payoff feedback after bond choice in gain domain	Trial payoff	Stick function
11. & 12. Bad payoff feedback after bond choice in gain domain	Trial payoff	Stick function
13. & 14. Good payoff feedback after stock choice in loss domain	Trial payoff	Stick function
15. & 16. Bad payoff feedback after stock choice in loss domain	Trial payoff	Stick function
17. & 18. Good payoff feedback after bond choice in loss domain	Trial payoff	Stick function
19. & 20. Bad payoff feedback after bond choice in loss domain	Trial payoff	Stick function
21. Stock estimation in gain domain	n.a.	Stick function
22. Stock estimation in loss domain	n.a.	Stick function
23. Current payoff status in gain domain	n.a.	Stick function
24. Current payoff status in loss domain	n.a.	Stick function
25. - 30. Six movement regressors	n.a.	n.a.
31. Constant	n.a.	n.a.

Abbreviations: n.a. = not applicable

Supplementary Table S3. Brain activation in the investing task (voxel-wise inclusion threshold, p(FWE)<0.05, k>10, n = 165), estimated using the GLM described in Supplementary Table S2.

Contrast name	Region	Laterality	MNI coordinates			Cluster size	T	Cluster p(FWE-corrected)
			x	y	z			
Stock vs. Bond choice (gain domain)	Sensorimotor cortex	R	39	-19	53	100	5.81	<0.001
	Visual cortex	L	-12	-97	8	15	5.19	0.003
Bond vs. Stock choice (gain domain)	No activation below significance threshold							
Stock vs. Bond choice (loss domain)	No activation below significance threshold							
Bond vs. Stock choice (loss domain)	Sensorimotor cortex	L	-36	-22	62	26	5.63	<0.001
Good vs. Bad payoff feedback after stock choice (gain domain)	Visual cortex	R	24	-94	8	963	8.33	<0.001
	Sensorimotor cortex	L	-42	-25	50	368	7.75	<0.001
	Ventromedial prefrontal cortex	L/R	-6	38	-10	179	6.72	<0.001
	Superior/ middle frontal gyrus /BA8	L	-15	29	50	70	6.55	<0.001
	Supplementary motor area	L	-3	-4	53	39	6.10	<0.001
	Thalamus	R	12	-10	-4	19	6.06	0.001
	Orbital part of the inferior frontal gyrus	R	30	32	-10	20	5.47	0.001
	Middle frontal gyrus/BA9	L	-30	41	32	10	5.26	0.005
Bad vs. Good payoff feedback after stock choice (gain domain)	No activation below significance threshold							
Good vs. Bad payoff feedback after stock choice (loss domain)	No activation below significance threshold							

Bad vs. Good payoff feedback after stock choice (loss domain)	No activation below significance threshold							
Good vs. Bad payoff feedback after bond choice (gain domain)	No activation below significance threshold							
Bad vs. Good payoff feedback after bond choice (gain domain)	No activation below significance threshold							
Good vs. Bad payoff feedback after bond choice (loss domain)	Visual cortex	R	24	-94	5	173	9.06	<0.001
	Visual cortex	L	-18	-94	-7	114	7.14	<0.001
	Anterior cingulate cortex	L	-9	35	2	10	5.21	0.005
Bad vs. Good payoff feedback after bond choice (loss domain)	No activation below significance threshold							
Good feedback after stock choice vs. Good feedback after bond choice (gain domain)	No activation below significance threshold							
Good feedback after bond choice vs. Good feedback after stock choice (gain domain)	No activation below significance threshold							
Good feedback after stock choice vs. Good feedback after bond choice (loss domain)	No activation below significance threshold							
Good feedback after bond choice vs. Good feedback after stock choice (loss domain)	Visual cortex	R	21	-97	8	22	6.22	0.001
Bad feedback after stock choice vs. Bad feedback after bond choice (gain domain)	No activation below significance threshold							

Bad feedback after bond choice vs. Bad feedback after stock choice (gain domain)	Sensorimotor cortex	L	-42	-22	50	126	5.95	<0.001
	Visual cortex	R	24	-94	5	20	5.8	0.001
	Angular gyrus	R	54	-52	23	41	5.75	<0.001
	Angular gyrus	L	-42	-64	35	87	5.38	<0.001
	Precuneus	R	3	-55	35	25	5.26	0.001
	Opercular and triangular part of the inferior frontal gyrus	L	39	17	29	37	5.19	<0.001
Bad feedback after stock choice vs. Bad feedback after bond choice (loss domain)	Insula	L	-42	5	-1	24	5.4	0.001
	Visual cortex	R	21	-97	5	21	5.37	0.001
Bad feedback after bond choice vs. Bad feedback after stock choice (loss domain)	Angular gyrus	L	-45	-64	38	34	5.23	<0.001
Good feedback after stock choice vs. Bad feedback after bond choice (gain domain)	Ventral striatum	L	-18	5	-7	12	6.57	0.003
	Ventral striatum	R	21	5	-10	19	6.19	0.001
	Ventromedial prefrontal cortex	R	9	38	-10	12	5.5	0.003
Bad feedback after bond choice vs. Good feedback after stock choice (gain domain)	Angular gyrus	R	57	-52	23	22	5.58	0.001
Good feedback after stock choice vs. Bad feedback after bond choice (loss domain)	No activation below significance threshold							
Bad feedback after bond choice vs. Good feedback after stock choice (loss domain)	No activation below significance threshold							
Good feedback after bond choice vs. Bad feedback after stock choice	Sensorimotor activation	L	-42	-22	59	65	5.87	<0.001
	Superior frontal	L	-15	32	50	10	5.18	0.005

(gain domain)	gyrus/BA8							
	Opercular part of the inferior frontal gyrus	R	39	14	35	11	5.00	0.004
	Precuneus	L/R	0	-55	38	15	4.93	0.002
Bad feedback after stock choice vs. Good feedback after bond choice (gain domain)	No activation below significance threshold							
Good feedback after bond choice vs. Bad feedback after stock choice (loss domain)	Angular gyrus	L	-51	-61	26	27	5.17	0.001
Bad feedback after stock choice vs. Good feedback after bond choice (loss domain)	No activation below significance threshold							

Region names based on the automatic anatomic labeling (aal) atlas. BA: Brodmann Area; L: Left; R: Right. Please note: the contrast of the parametrical modulators vs. baseline did not yield any activation below the significance threshold and are thus not included in the table.

Supplementary Table S4. Mean extracted weighted beta estimates of non-active stock traders (NAST, n = 109) and active stock traders (AST, n = 48). Standard error in parentheses. The only two significant two-sample t-test results ($p<0.05$, uncorrected) are shown in bold and were $p=0.0264$ for the left AI (L AI) and $p=0.0072$ for the right AI (R AI).

Contrast number	Contrast description	Region of interest	NAST Mean (SE)	AST Mean (SE)
1	Stock vs. Bond choice (gain domain)	**L AI**	**-0.06 (±0.058)**	**-0.31 (±0.103)**
		**R AI**	**0.02 (±0.066)**	**-0.31 (±0.105)**
		vmPFC	0.08 (±0.089)	0.24 (±0.124)
		VS	0.20 (±0.073)	0.07 (±0.119)
		R FFA	0.05 (±0.085)	0.10 (±0.102)
2	Stock vs. Bond choice (loss domain)	L AI	0.07 (±0.056)	-0.02 (±0.087)
		R AI	0.07 (±0.060)	0.02 (±0.066)
		vmPFC	-0.08 (±0.055)	0.03 (±0.096)
		VS	0.12 (±0.057)	0.07 (±0.096)
3	Good vs. Bad payoff feedback after stock choice (gain domain)	L AI	0.18 (±0.102)	0.29 (±0.178)
		R AI	0.10 (±0.115)	0.14 (±0.133)
		vmPFC	0.72 (±0.136)	0.36 (±0.188)
		VS	0.50 (±0.134)	0.32 (±0.167)
4	Good vs. Bad payoff feedback after stock choice (loss domain)	L AI	-0.29 (±0.125)	0.02 (±0.172)
		R AI	-0.04 (±0.129)	0.04 (±0.172)
		vmPFC	0.39 (±0.174)	-0.20 (±0.233)
		VS	0.12 (±0.161)	0.24 (±0.197)
5	Good vs. Bad payoff feedback after bond choice (gain domain)	L AI	-0.07 (±0.143)	-0.25 (±0.246)
		R AI	-0.16 (±0.152)	-0.03 (±0.239)
		vmPFC	-0.09 (±0.214)	0.13 (±0.280)
		VS	0.17 (±0.191)	0.58 (±0.307)
6	Good vs. Bad payoff feedback after bond choice (loss domain)	L AI	0.25 (±0.107)	0.08 (±0.172)
		R AI	0.11 (±0.120)	0.29 (±0.146)
		vmPFC	0.42 (±0.134)	0.18 (±0.292)
		VS	0.24 (±0.130)	0.33 (±0.222)
7	Good feedback after stock choice vs. Good feedback after bond choice (gain domain)	L AI	0.27 (±0.114)	0.42 (±0.238)
		R AI	0.21 (±0.137)	0.08 (±0.220)
		vmPFC	0.65 (±0.209)	0.19 (±0.290)
		VS	0.35 (±0.196)	0.04 (±0.293)
8	Good feedback after stock choice vs. Good feedback after bond choice (loss domain)	L AI	-0.06 (±0.113)	-0.01 (±0.205)
		R AI	0.23 (±0.135)	-0.04 (±0.213)
		vmPFC	0.01 (±0.167)	-0.12 (±0.271)
		VS	0.13 (±0.142)	0.05 (±0.257)
9	Bad feedback after stock choice vs. Bad feedback after bond choice (gain domain)	L AI	0.02 (±0.133)	-0.11 (±0.200)
		R AI	-0.06 (±0.138)	-0.08 (±0.166)
		vmPFC	-0.16 (±0.151)	-0.04 (±0.239)
		VS	0.03 (±0.162)	0.30 (±0.258)

10	Bad feedback after stock choice vs. Bad feedback after bond choice (loss domain)	L AI	0.49 (±0.134)	0.05 (±0.183)
		R AI	0.38 (±0.138)	0.20 (±0.143)
		vmPFC	0.04 (±0.183)	0.26 (±0.246)
		VS	0.26 (±0.188)	0.14 (±0.232)
11	Good feedback after stock choice vs. Bad feedback after bond choice (gain domain)	L AI	0.21 (±0.126)	0.18 (±0.245)
		R AI	0.05 (±0.135)	0.06 (±0.192)
		vmPFC	0.56 (±0.161)	0.33 (±0.212)
		VS	0.53 (±0.155)	0.62 (±0.239)
12	Good feedback after stock choice vs. Bad feedback after bond choice (loss domain)	L AI	0.20 (±0.115)	0.07 (±0.186)
		R AI	0.34 (±0.124)	0.24 (±0.178)
		vmPFC	0.43 (±0.167)	0.06 (±0.270)
		VS	0.37 (±0.170)	0.38 (±0.223)
13	Good feedback after bond choice vs. Bad feedback after stock choice (gain domain)	L AI	-0.09 (±0.129)	-0.13 (±0.248)
		R AI	-0.11 (±0.146)	0.05 (±0.208)
		vmPFC	0.07 (±0.211)	0.17 (±0.278)
		VS	0.15 (±0.184)	0.28 (±0.280)
14	Good feedback after bond choice vs. Bad feedback after stock choice (loss domain)	L AI	-0.24 (±0.136)	0.03 (±0.198)
		R AI	-0.27 (±0.144)	0.09 (±0.197)
		vmPFC	0.38 (±0.176)	-0.08 (±0.283)
		VS	-0.01 (±0.172)	0.19 (±0.247)

AI: anterior insula; FFA: fusiform face area; L: left; R: right; vmPFC: ventromedial prefrontal cortex; VS: ventral striatum.

Supplementary Table S5. Descriptive statistics of the variables that were used to create the indices of risk optimism and risk tolerance.

Risk optimism	*Mean*	*SD*	*Min*	*Max*	*N*	*Q#*
Self-assessment of financial knowledge	*4.4*	*1.3*	*1*	*7*	198	32a
Self-assessment of mathematical abilities	*4.8*	*1.3*	*1*	*7*	197	32b
Believing that speculation is a useful way to increase money	*2.0*	*1.0*	*1*	*4*	196	30f
Trusting your own financial expertise	*2.5*	*0.8*	*1*	*4*	196	30c
Thoroughly checking the theoretical probabilities before making financial investments to avoid mistakes	*3.2*	*0.8*	*1*	*4*	196	30b
Behavioural risk optimism in the investing paradigm	*0.6*	*5.5*	*-18.8*	*19.4*	189	n.a.
Risk Optimism Index (ROI)	*0.0*	*1.4*	*-3.1*	*3.7*	195	n.a.
Risk tolerance						
Self-assessment of financial risk taking	*3.4*	*1.4*	*1*	*7*	196	29b
Self-assessment of risk taking when trusting strangers	*4.0*	*1.5*	*1*	*7*	196	29f
Risk taking in the hypothetical lottery question	*1.4*	*1.4*	*0*	*5*	198	38
Liking the excitement of risks	*2.2*	*0.9*	*1*	*4*	196	30a
Feeling that the urge to win outweighs the fear to lose	*2.1*	*0.9*	*1*	*4*	196	30d
Feeling psychologically pressured due to financial risks	*2.3*	*0.9*	*1*	*4*	195	30e
Behavioural risk taking in the investing paradigm	*57.5*	*26.5*	*0.0*	*100.0*	189	n.a.
Behavioural risk taking in the stock allocation task	*11.2*	*4.4*	*0.4*	*23.0*	193	n.a.
Risk Tolerance Index (RTI)	*0.0*	*1.4*	*-2.7*	*3.5*	187	n.a.

Q# = number in financial, risk preference, and personality questionnaire, provided as a Supplementary Document. n.a. = not applicable

Supplementary Table S6. Logistic regression results of active stock trading including risk optimism-related measures and the Risk Optimism Index (ROI).

	Active stock trading						
Self-assessment of financial knowledge	4.09*** (0.274)						
Self-assessment of mathematical abilities		-0.26 (0.120)					
Believing that speculation is a useful way to increase money			6.24*** (0.882)				
Trusting your own financial expertise				3.54*** (0.477)			
Thoroughly checking the theoretical probabilities before making financial investments to avoid mistakes					-1.78* (0.137)		
Behavioural risk optimism in the investing paradigm						0.92 (0.030)	
Risk Optimism Index (ROI)							5.30*** (0.340)
N	198	197	196	196	196	189	196
Pseudo R^2	0.0844	0.0003	0.2307	0.0586	0.0134	0.0037	0.1609

Significance levels: *p<0.1, **p<0.05, ***p<0.001. Z-values are shown with standard errors in parentheses.

Supplementary Table S7. Logistic regression results of active stock trading including risk tolerance-related measures and the Risk Tolerance Index (RTI).

	Active stock trading								
Self-assessment of financial risk taking	6.37*** (0.435)								
Self-assessment risk taking when trusting strangers		-1.96* (0.084)							
Risk taking in the hypothetical lottery question			1.67* (0.135)						
Liking the excitement of risks				2.22* (0.268)					
Feeling that the urge to win outweighs the fear to lose					3.76*** (0.398)				
Feeling psychologically pressured due to financial risks						-2.65** (0.114)			
Behavioural risk taking in the investing paradigm							1.79* (0.007)		
Behavioural risk taking in the allocation paradigm								-0.47 (0.036)	
Risk Tolerance Index (RTI)									5.05*** (0.243)
N	196	196	198	196	196	195	189	193	186
Pseudo R^2	0.2531	0.0166	0.0117	0.0216	0.0653	0.0317	0.0148	0.0009	0.1363

Significance levels: *p<0.1, **p<0.05, ***p<0.001. Z-values are shown with standard errors in parentheses.

Supplementary Table S8. The principal component analysis results using four risk optimism variables (n = 196). The first component (CP1) was used as the Risk Optimism Index (ROI).

	Factor loading			
	CP1	CP2	CP3	CP4
Self-assessment of financial knowledge	0.639	0.155	-0.284	0.698
Believing that speculation is a useful way to increase money	0.424	-0.470	0.774	0.032
Trusting your own financial knowledge	0.642	0.183	-0.211	-0.714
Thoroughly checking the theoretical probabilities before to avoid mistakes	-0.021	0.850	0.525	-0.044
Eigenvalue	1.92	1.12	0.68	0.28
Proportion %	47.9	28.1	17.1	0.1

Supplementary Table S9. The principal component analysis results using seven risk tolerance variables (n = 186). The first component (CP1) was used as the Risk Tolerance Index (RTI).

	Factor loading						
	CP1	CP2	CP3	CP4	CP5	CP6	CP7
Self-assessment of financial risk taking	0.554	0.125	0.089	-0.140	-0.008	0.108	0.799
Self-assessment of risk taking when trusting strangers	0.100	-0.708	-0.180	0.384	0.366	0.366	0.077
Risk taking in the hypothetical lottery question	0.394	-0.051	-0.218	0.617	-0.506	-0.388	-0.085
Feeling that the urge to win outweighs the fear to lose	0.453	-0.049	0.229	-0.072	0.620	-0.528	-0.267
Liking the excitement of risks	0.466	0.024	0.452	-0.086	-0.267	0.530	-0.467
Feeling psychologically pressured due to financial risks	-0.310	0.102	0.740	0.539	0.082	-0.042	0.218
Behavioural risk taking in the investing paradigm	0.094	0.684	-0.328	0.387	0.384	0.33	-0.108
Eigenvalue	2.22	1.23	0.92	0.85	0.73	0.64	0.42
Proportion %	31.7	17.5	13.1	12.1	10.4	9.1	6.0

Supplementary Table S10. Mediation models investigating the mediation of the brain activation in the left and right anterior insula (AI, both independent variables) to active stock trading (AST, dependent variable) through the risk tolerance and optimism indices (RTI and ROI, both mediators), as well as the financial Risk Seeking and Preference Index (RSPI, also a mediator). The Sobel-Goodman (SG) Mediation test was used with subsequent bootstrapping of the effect (all n = 157, seed set at 10, 10,000 repetitions). An effect is considered significant if the confidence interval does not include the null hypothesis (i.e. zero is not included).

Pathway (IV → MV → DV)	a*b (indirect effect)	c (total effect)	c' (direct effect)	mediation[1] (indirect to total)
l.AI → RTI → AST	-0.08 (-0.145 to -0.028)	-0.13 (-0.240 to -0.001)	-0.05 (-0.157 to 0.075)	0.64 (≥0.177)
l.AI → ROI → AST	-0.07 (-0.116 to -0.035)	-0.13 (-0.240 to -0.001)	-0.06 (-0.162 to 0.064)	0.56 (≥0.229)
r.AI → RTI → AST	-0.07 (-0.121 to -0.032)	-0.14 (-0.238 to -0.030)	-0.07 (-0.156 to 0.029)	0.52 (≥0.250)
l.AI → RSPI → AST	-0.11 (-0.176 to -0.050)	-0.13 (-0.240 to -0.001)	-0.02 (-0.123 to 0.098)	0.86 (≥0.353)
r.AI → RSPI → AST	-0.08 (-0.137 to -0.030)	-0.14 (-0.238 to -0.030)	-0.06 (-0.135 to 0.031)	0.59 (≥0.305)

Observed coefficients are shown with 95% (bias-corrected and accelerated) confidence intervals in parentheses.

[1]Please note that the full confidence intervals for the mediation (indirect to total) effect were not included, given that this measure can exceed the reasonable values (i.e. ≤1.00) and has to be taken with caution[1]. We thus only included the fact that the 95% confidence interval remains positive and does not include zero, thus implying a significant mediation effect. Please note that in all cases, a switch of the mediator and independent variables resulted in no significant mediation.

Supplementary Table S11. Pairwise correlations (uncorrected) of the latent variables measured in the experiment, meant as a sanity check. Here: Intelligence and the stock assessment error in the investing paradigm.

	Figural intelligence	Numerical intelligence	Verbal intelligence	Stock assessment error
Figural intelligence		0.432***<br>196	0.278**<br>196	-0.216**<br>189
Numerical intelligence			0.432***<br>196	-0.153**<br>189
Verbal intelligence				-0.308***<br>189
Stock assessment error				

Coefficients r and number of observations shown. Significance levels: *p<0.1, **p<0.05, ***p<0.001.

Supplementary Table S12. The principal component analysis results using the significant variables from both the risk optimism and the risk tolerance category (n = 186). The first component (CP1) was used as the financial Risk Seeking and Preference Index (RSPI).

	Factor loading										
	CP1	CP2	CP3	CP4	CP5	CP6	CP7	CP8	CP9	CP10	CP11
Self-assessment of financial risk taking	0.479	-0.120	-0.084	0.047	0.109	-0.093	0.008	0.027	0.191	-0.779	-0.287
Self-assessment of risk taking when trusting strangers	0.007	-0.371	0.586	-0.233	-0.220	0.404	0.275	0.041	0.420	0.007	0.016
Risk taking in the hypothetical lottery question	0.278	-0.161	0.104	0.502	-0.316	0.471	-0.402	0.030	-0.374	-0.018	0.105
Feeling that the urge to win outweighs the fear to lose	0.300	-0.342	-0.117	0.195	0.156	-0.071	0.691	0.228	-0.363	0.226	-0.023
Liking the excitement of risks	0.358	-0.190	-0.041	0.034	0.452	0.098	-0.147	-0.661	0.202	0.345	-0.034
Feeling psychologically pressured due to financial risks	-0.246	0.097	-0.221	0.014	0.595	0.643	0.015	0.307	0.060	-0.118	0.024
Behavioural risk taking in the investing paradigm	0.132	0.246	-0.596	-0.027	-0.482	0.329	0.317	-0.214	0.272	0.039	0.063
Self-assessment of financial knowledge	0.336	0.469	0.289	-0.152	0.141	0.028	0.175	-0.091	-0.159	-0.175	0.668
Believing that speculation is a useful way to increase money	0.418	0.006	-0.123	-0.005	0.017	-0.158	-0.285	0.590	0.445	0.346	0.193
Trusting your own financial knowledge	0.282	0.548	0.265	-0.130	-0.011	0.165	0.044	0.093	-0.143	0.238	-0.646
Thoroughly checking the theoretical probabilities before to avoid mistakes	-0.172	0.282	0.227	0.782	0.071	-0.131	0.224	-0.047	0.391	0.001	-0.013
Eigenvalue	2.99	1.67	1.13	0.95	0.91	0.86	0.70	0.64	0.53	0.36	0.25
Proportion %	27.2	15.2	10.2	8.7	8.3	7.8	6.4	5.8	4.9	3.3	2.3

Supplementary Table S13. Linear and multiple regression results of active stock trading including activation in the left anterior insula and the financial Risk Seeking and Preference Index (RSPI). Coefficients are shown with t-statistics in parentheses.

	Active stock trading			
Left AI (stock > bond choice, gain domain)	-0.13	-0.13	-0.10	0.0002
	(-2.24)**	(-2.39)**	(-1.93)*	(0.00)
Household income (after taxes)		0.10	0.09	0.06
		(4.72)***	(4.54)***	(3.72)***
Having financial liabilities		-0.11	-0.12	-0.11
		(-1.54)	(-1.60)	(-1.77)*
Years of education			0.03	0.02
			(1.78)*	(1.70)*
Financial literacy			0.04	-0.03
			(0.41)	(-0.35)
Debt literacy			-0.01	-0.10
			(-0.16)	(-1.57)
Numeracy			-0.10	-0.04
			(-0.81)	(-0.40)
Verbal intelligence			0.003	0.004
			(0.64)	(0.98)
Numerical intelligence			0.0003	0.001
			(0.07)	(0.26)
Figural intelligence			-0.01	-0.01
			(-1.99)**	(-2.08)**
Financial Risk Seeking and Preference Index (RSPI)				0.14
				(7.86)***
N	157	157	157	157
Adjusted R^2	0.03	0.15	0.16	0.41

Significance levels: *p<0.1, **p<0.05, ***p<0.001.

Supplementary Table S14. Linear and multiple regression results of active stock trading including activation in the right anterior insula and the financial Risk Seeking and Preference Index (RSPI). Coefficients are shown with t-statistics in parentheses.

	Active stock trading			
Right AI (stock > bond choice, gain domain)	-0.14	-0.15	-0.14	-0.06
	(-2.72)**	(-3.14)**	(-2.85)**	(-1.41)
Household income (after taxes)		0.10	0.09	0.07
		(4.84)***	(4.68)***	(3.88)***
Having financial liabilities		-0.13	-0.14	-0.12
		(-1.83)*	(-1.87)*	(-1.86)*
Years of education			0.03	0.02
			(1.90)*	(1.79)*
Financial literacy			0.03	-0.03
			(0.39)	(-0.35)
Debt literacy			-0.01	-0.09
			(-0.12)	(-1.43)
Numeracy			-0.08	-0.03
			(-0.69)	(-0.26)
Verbal intelligence			0.004	0.004
			(0.76)	(0.97)
Numerical intelligence			0.0004	0.001
			(0.11)	(0.24)
Figural intelligence			-0.01	-0.007
			(-1.96)*	(-1.95)*
Financial Risk Seeking and Preference Index (RSPI)				0.13
				(7.66)***
N	157	157	157	157
Adjusted R^2	0.04	0.17	0.19	0.42

Significance levels: *p<0.1, **p<0.05, ***p<0.001.
